# ISG15 conjugation to proteins on nascent DNA mitigates DNA replication stress

**DOI:** 10.1038/s41467-022-33535-y

**Published:** 2022-10-10

**Authors:** Christopher P. Wardlaw, John H. J. Petrini

**Affiliations:** Molecular Biology Program, Memorial Sloan-Kettering Cancer Center, New York, NY USA

**Keywords:** Stalled forks, DNA damage response

## Abstract

The pathways involved in suppressing DNA replication stress and the associated DNA damage are critical to maintaining genome integrity. The Mre11 complex is unique among double strand break (DSB) repair proteins for its association with the DNA replication fork. Here we show that Mre11 complex inactivation causes DNA replication stress and changes in the abundance of proteins associated with nascent DNA. One of the most highly enriched proteins at the DNA replication fork upon Mre11 complex inactivation was the ubiquitin like protein ISG15. Mre11 complex deficiency and drug induced replication stress both led to the accumulation of cytoplasmic DNA and the subsequent activation of innate immune signaling via cGAS-STING-Tbk1. This led to *ISG15* induction and protein ISGylation, including constituents of the replication fork. ISG15 plays a direct role in preventing replication stress. Deletion of *ISG15* was associated with replication fork stalling, tonic ATR activation, genomic aberrations, and sensitivity to aphidicolin. These data reveal a previously unrecognized role for ISG15 in mitigating DNA replication stress and promoting genomic stability.

## Abstract (web summary)

DNA replication stress can result in genome instability. Here the authors show that the ubiquitin like modifier protein, ISG15, important during the innate immune response, acts at replication forks to mitigate DNA replication stress.

## Introduction

To preserve genomic integrity, all living organisms have evolved complex pathways to sense, signal, and repair damaged DNA. Collectively this network of pathways comprises the DNA damage response (DDR)^1^. A key component of the DDR is the highly conserved Mre11 complex, consisting of Rad50, Mre11 and Nbs1. The Mre11 complex is a sensor of DSBs and is required for activation of ATM, an apical kinase in the DDR network^2^. The complex also specifies endo- and exonuclease activities, and is required for both homologous recombination (HR) and nonhomologous end joining (NHEJ)^3^. The importance of the complex in maintaining genome integrity is underscored by the occurrence of mutations in *Rad50*, *Mre11* or *Nbs1* in roughly five percent of solid tumors^4,5^. Congenitally acquired mutations in Mre11 complex encoding genes underlie chromosome instability disorders such as Nijmegen Breakage Syndrome and the ataxia telangiectasia like disorder ATLD^6,7^.

Several lines of evidence suggest the Mre11 complex is integral to DNA replication. Yeast cells devoid of the Mre11 complex show a sixty-fold increase in gross chromosomal rearrangements (GCRs) over *Rad51-Ku70* double mutants which are completely double strand break repair deficient. The levels of GCR in Mre11 complex deficient cells is similar to those seen in cells deficient for Rad27 (Fen1) and other replication proteins^8^. In higher eukaryotes, the Mre11 complex is essential in cycling cells, but is dispensable in quiescent cells^9^. The complex associates with chromatin during S-phase, co-localizes with PCNA, and is enriched on newly synthesized DNA in human cells^10–12^. These observations indicate the Mre11 complex functions at the DNA replication fork in a manner distinct from other DSB repair proteins.

Interferon stimulated gene 15 (*ISG15*) encodes a 18 kDa ubiquitin-like modifier consisting of two ubiquitin domains separated by a flexible linker^13,14^. ISG15 is covalently conjugated to target proteins via its C-terminal LRLRGG motif in a process analogous to the conjugation of Ubiquitin and Sumo called ISGylation^15–17^. *ISG15* expression is induced by type I interferon signaling, cytoplasmic nucleic acid sensing pathways and other cellular stresses^18^. Most of the information on ISG15 has come from studying viral infection, where ISGylation of viral and host proteins limits viral replication^19,20^. However, recent studies have shown ISG15 and ISGylation is involved in the response to other forms of cellular stress and may act to regulate a wide range of cellular functions and processes^19^. Full understanding of ISGylation targets and the functions ISG15 regulates in various contexts remains to be established.

Given the close association of the Mre11 complex with the replication fork^10–12^, we examined the effect(s) of Mre11 complex deficiency on replication fork progression and composition. We find that loss of the Mre11 complex profoundly alters the protein landscape on nascent DNA, with scores of proteins being both depleted and enriched at the fork. Mre11 complex depletion also led to Chk1 phosphorylation, replication fork stalling and the activation of the cGAS-STING-Tbk1 cytoplasmic DNA sensing pathway. The induction of cGAS signaling led to *ISG15* expression and protein ISGylation, including that of DNA replication associated proteins. Those modifications play a role in DNA replication, as deletion of ISG15 altered the constituents of the replication fork, caused ATR activation, replication fork stalling and spontaneous chromosome aberrations. The data therefore suggest that conjugation of ISG15 to proteins at the replication fork suppresses DNA replication stress and promotes genome stability.

## Results

**Fig. 1:**
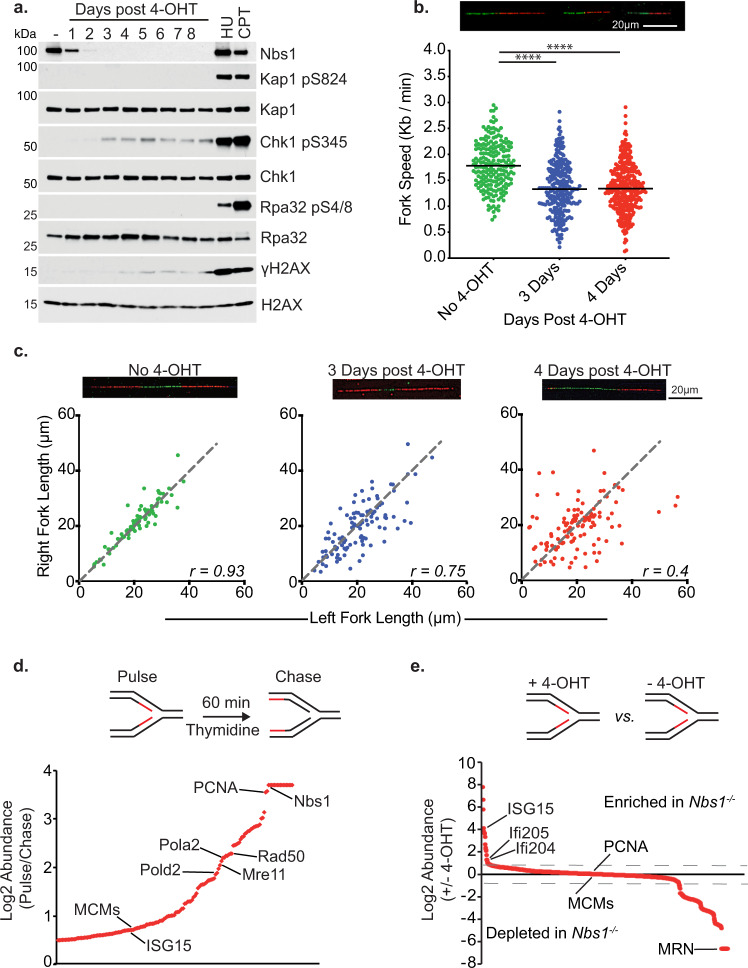
Deletion of *Nbs1* Impacts DNA Replication. **a** *Nbs1*^*f/f*^ MEFs treated with 4-OHT for 48 h. Whole cell extracts were taken at the indicated time points post 4-OHT addition for western blotting. Extracts from 2 mM HU and 2 μM CPT (2 h) treated cells served as controls for antibody specificity. **b** DNA combing analysis of *Nbs1*^*f/f*^ at the indicated timepoints post 4-OHT addition. IdU = green fiber, CldU = Red fiber. CldU fiber length was measured, and fork speed calculated. Combined data from three independent experiments (No 4-OHT *n* = 207, 3 days *n* = 250, 4 days *n* = 329). Median velocity shown. Two Tailed Mann–Whitney test ****= *p* < 0.0001. **c** As in b. except Left-Right ratios of CldU fibers initiating from the same origin were measured. Combined data from three independent experiments. (No 4-OHT *n* = 82, 3 days *n* = 106, 4 days *n* = 122). Pearson coefficient (*r*) shown. **d** Pulse-Chase iPOND-SILAC-MS showing mean Log2 ratio of pulse *vs*. chase protein intensities from three independent experiments. Proteins with a Log2 ratio of at least 0.5 plotted in order of ascending ratio. **e** iPOND-SILAC-MS from *Nbs1*^*f/f*^ cells 3 days post 4-OHT addition *vs*. no 4-OHT. Proteins identified in at least two out of three experiments were ranked by mean Log2 ratio. Boundaries for enriched and depleted proteins represented by dotted lines at inflection points within the data. Source data are provided with this paper.

To examine the effect of the Mre11 complex on the composition and progression of the replication fork, we used a cell line in which deletion of *Nbs1* is induced by the Cre recombinase. The system comprises SV40-immortalized mouse embryonic fibroblasts (MEFs) in which exon six of *Nbs1* is flanked by *loxP* sites and a *Cre*^*ERT*^ cassette is constitutively expressed (*Nbs1*^*f/f*^) (Supplementary Fig. 1a)^21,22^. Addition of tamoxifen (4-OHT) leads to Cre^ERT^ nuclear localization, the deletion of *Nbs1* exon 6, and the loss of all detectable Nbs1 within three days (referred to as *Nbs1*^*−/−*^) (Fig. 1a). Deletion of *Nbs1* is associated with retention of Mre11 and Rad50 in the cytoplasm which blocks the complex’s nuclear functions and causes cell death between six and ten days post 4-OHT addition^22^.

### The Mre11 complex mitigates the outcomes of DNA replication stress

For the purposes of this study, DNA replication stress is defined by two parameters. First is the activation of the ATR kinase which phosphorylates several targets including Chk1 and H2AX, and governs S-phase checkpoint activation^23^. At 3 days post 4-OHT addition, the first time point at which Nbs1 is undetectable, Chk1 and H2AX phosphorylation were observed, consistent with ATR activation (Fig. 1a). No Kap1 S824 or Rpa S4/8 phosphorylation, markers of ATM activation, was detected upon *Nbs1* deletion (Fig. 1a), ruling out ATM dependent phosphorylation of Chk1 and H2AX in this setting.

The second parameter used to define DNA replication stress is the perturbation of replication fork progression. To test the hypotheses that ATR activation upon *Nbs1* deletion was associated with impaired replication fork progression, DNA combing was performed at 3- and 4-days post 4-OHT addition (Fig. 1a, b). Cells were pulsed with IdU for 30 min followed by a 30-min CldU pulse. The length of the CldU tracts was then measured as an indicator of fork speed. Upon *Nbs1* deletion a 27.8% reduction in median replication fork speed was observed, indicating a replication defect in *Nbs1* deficient cells (Fig. 1b). Upon *Nbs1* depletion, an increase in asymmetric sister CldU tracts was also observed in the *Nbs1*^*f/f*^ cells after 4-OHT addition (No 4-OHT *r* = 0.93; 3 days post *r* = 0.75; 4 days post *r* = 0.4). This outcome indicates *Nbs1* deficiency results in DNA replication fork stalling (Fig. 1c). EDU incorporation monitored by flow cytometry showed a 30% reduction in EDU intensity at 3 days post 4-OHT addition, without any change in cell cycle distribution (Supplementary Fig. 1b–d), consistent with the reduced replication fork speed and increased fork stalling observed (Fig. 1b, c). Taken together, these data show that the Mre11 complex prevents DNA replication stress, and indicate that the complex is integral to DNA replication fork progression and stability.

### Mre11 complex deficiency alters the protein composition at the replication fork

Isolation of Proteins on Nascent DNA (iPOND)^24^ experiments were carried out in the *Nbs1*^*f/f*^ system to examine the effect of Mre11 complex depletion on replication fork associated proteins. First, pulse-chase iPOND followed by SILAC mass spectrometry was performed to confirm that the Mre11-complex localizes to replication forks in MEFs, as it does in human cells (Fig. 1d, Supplementary Fig. 2a, Supplementary Data 1)^12,25^. Cells were pulsed for 10 min with EdU and either immediately fixed (nascent DNA) or chased with thymidine for 60 min before fixation. iPOND was then performed as previously described^26^ (Fig. 1d). The abundance of each of the six MCM replicative helicase components were assessed as quality control markers and found to be equivalent (Supplementary Fig. 2b)^27^.

All components of the Mre11 complex were highly enriched on nascent DNA compared to the chase sample, showing comparable enrichments to known replisome components such as PCNA and the replicative polymerases (Fig. 1d and Supplementary Data 1). Pulse-chase iPOND followed by western blotting confirmed this result, showing that the Mre11 complex localizes to the replication fork in MEFs (Supplementary Fig. 2c). Neither homologous recombination proteins such as Rad51 or Rad52, nor non-homologous end joining proteins such as Ku or DNA-Pkcs were enriched on nascent DNA. This argues that the localization of the Mre11 complex to nascent DNA is not due to the presence of broken replication forks (Supplementary Data 1).

To determine whether Mre11 complex deficiency affects replisome composition, iPOND SILAC-mass-spectrometry from *Nbs1*^*f/f*^ cells with or without 4-OHT addition was carried out (Fig. 1e and Supplementary Fig. 2d) (hereafter, *Nbs1*^*f/f*^ without 4-OHT is designated *WT* and three days after 4-OHT, *Nbs1*^*−/−*^). Comparable enrichments of MCM components were again observed (Supplementary Fig. 2e). Deletion of *Nbs1* led to changes in protein abundance at the replication fork. 181 proteins were enriched (fold change ≥1.3) on nascent DNA in *Nbs1*^*−/−*^ compared to *WT* cells and 176 were depleted (fold change ≤0.7) (Fig. 1e and Supplementary Data 2). Taken together with the results from the pulse-chase iPOND, these data show the Mre11 complex is enriched at the DNA replication fork, and that loss of Nbs1 leads to substantial changes in the constituent proteins associated with nascent DNA. It is probable these changes represent both a cause and a consequence of the replication stress observed in Nbs1 deficient cells.

### Mre11 complex deficiency induces ISG15 expression and ISGylation

Upon *Nbs1* deletion we saw an increase in innate immune response proteins associated with nascent DNA (Fig. 1e and Supplementary Data 2). The most highly enriched of these proteins was the ubiquitin like modifier protein ISG15 (38.5-fold average enrichment relative to *WT* cells*)* (Fig. 1e and Supplementary Data 2). ISG15 was also enriched 1.57-fold on nascent DNA compared to bulk chromatin in the pulse-chase iPOND-SILAC experiment carried out in wild type MEFs, a similar enrichment to that seen for the MCM complex (Supplementary Data 1, and Fig. 1d). Thus, ISG15 enrichment is occurring primarily at the replication fork.

**Fig. 2:**
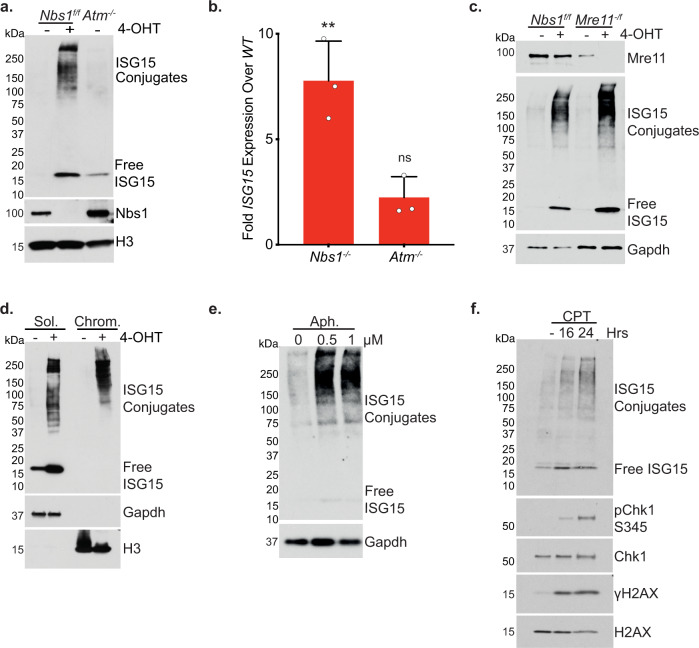
Loss of Mre11 Complex Function Induces ISG15 and ISGylation. **a** Western blot of whole cell extracts taken three days post 4-OHT addition or from untreated control. **b** ISG15 expression measured by qPCR. *Nbs1*^*−/−*^ is three days post 4-OHT addition. Mean of three independent experiments shown with SD. Two-tailed unpaired *t*-test, ns= not significant, **= *p* 0.0034. **c** Western blot of whole cell extracts from cells with or without induction of *Nbs1* or *Mre11* deletion (3- and 7-days post 4-OHT, respectively). **d** Cellular fractionation and western blot from *Nbs1*^*f/f*^ MEFs taken three days post 4-OHT addition or from untreated control. **e** Western blot analysis of whole cell extracts taken 16 h after aphidicolin (Aph) addition at the indicated concentrations. **f** Western blot analysis of whole cell extracts taken at the indicated timepoints after 0.25 μM Camptothecin (CPT) addition. Source data are provided with this paper.

Free ISG15 and ISG15 conjugated proteins were undetectable in whole cell extracts prior to 4-OHT addition in *Nbs1*^*f/f*^ cells, but were clearly evident at three days post 4-OHT addition (Fig. 2a). Accordingly, RT-qPCR showed an 8-fold increase in *ISG15* transcripts upon *Nbs1* deletion (Fig. 2b). The increases in *ISG15* expression and ISGylation were also seen in *Nbs1*^*-/F*^ and *Mre11*^*-/F*^ MEFs after 4-OHT addition, demonstrating that *ISG15* induction and ISGylation are a general consequence of Mre11 complex inactivation. (Fig. 2c and Supplementary Fig. 3a). Those outcomes are not a consequence of SV40 immortalization, as it also occurred upon *Nbs1* deletion in *p53*^*−/−*^ immortalized *Nbs1*^*f/f*^ MEFs, nor is it a response to *CRE*^*ERT*^ expression or its localization and activity in the nucleus upon 4-OHT addition (Supplementary Fig. 3b–e).

Cellular fractionation and western blotting of soluble (cytoplasmic) and insoluble (chromatin) fractions showed that chromatin bound proteins were ISGylated upon *Nbs1* deletion. In contrast to the cytoplasmic fraction, no free (unconjugated) ISG15 was detected in the chromatin fraction, supporting the interpretation that ISG15 is conjugated to proteins associated with nascent DNA (Fig. 2d).

ATM deficient cells have previously been shown to have constitutively induced *ISG15* and ISGylated proteins^28,29^. ATM activation is abolished by Mre11 complex deficiency; however, the levels of ISG15 and ISGylation are much greater upon *Nbs1* deletion than in *ATM*^*−/−*^ cells, indicating that a defect in ATM activation is not a primary cause of the ISGylation in Mre11 complex deficient cells (Fig. 2a, b).

### Cytoplasmic DNA underlies ISG15 induction in Mre11 complex deficient cells

**Fig. 3:**
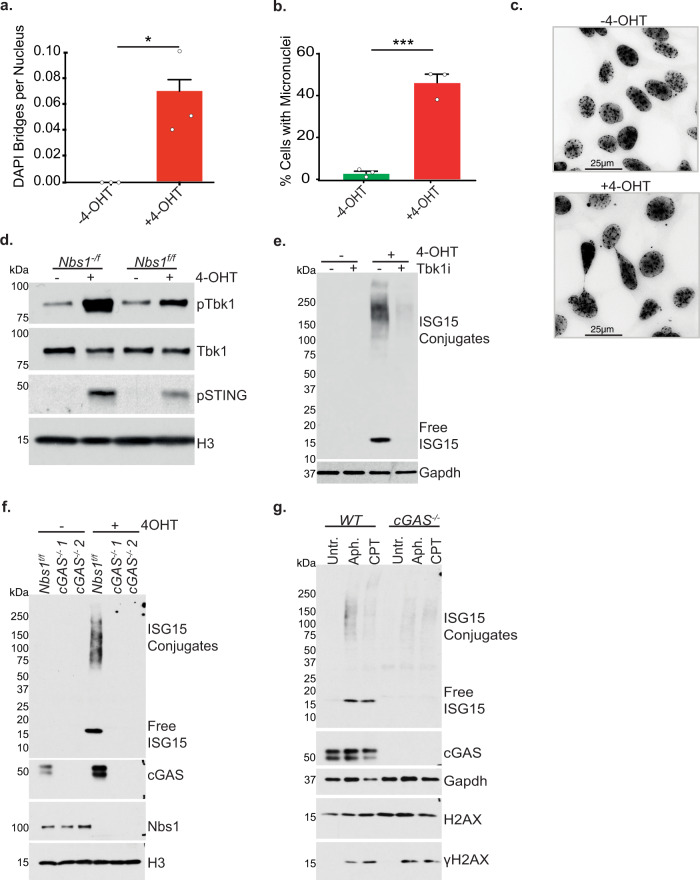
Cytoplasmic DNA underlies ISG15 induction Upon *Nbs1* Deletion. **a** DAPI stained DNA bridges per nucleus for *Nbs1*^*f/f*^ MEFs three days post 4-OHT addition. Mean of three independent experiments with SEM. Two-tailed unpaired *t*-test was used. * = *p* 0.0270. **b** The percentage of *Nbs1*^*f/f*^ MEFs 3 days post 4-OHT addition cells with micronuclei. Mean of three independent experiments with SEM. Two-tailed unpaired *t*-test was used. *** = *p* 0.0005 **c** Example images for the data shown in a and b. Between 105 and 152 cells were counted per condition per biological repeat (−4-OHT *n* = 371, +4-OHT *n* = 416). **d** Western blot of whole cell extracts from *Nbs1*^*f/f*^ and *Nbs1*^*-/f*^ with or without 4-OHT induced *Nbs1* deletion. **e** Western blot of whole cell extracts with or without 4-OHT induced *Nbs1* deletion, in the presence or absence of 5 μM MRT67307 Tbk1 inhibitor. **f** Western blot of whole cell extracts taken from *Nbs1*^*f/f*^ or *Nbs1*^*f/f*^ *cGAS*^*−/−*^ with or without 4-OHT induced *Nbs1* deletion. Two independent *Nbs1*^*f/f*^ *cGAS*^*−/−*^ clones shown. **g** Western blot of whole cell extracts from the indicated MEFs, with or without 24 h of 1 μM aphidicolin (Aph) or 0.25 μM camptothecin (CPT) treatment. Source data are provided with this paper.

DNA replication stress is commonly associated with the accumulation of extranuclear DNA including DNA bridges and micronuclei (Supplementary Fig. 4a–c)^30^. These structures activate cGAS-STING-Tbk1 signaling^31–33^ resulting in *ISG15* expression and ISGylation (Fig. 2e, f). It is therefore likely that the replication stress associated with Nbs1 deficiency (Fig. 1a–c) is the primary cause of ISGylation in these cells. Indeed, Nbs1 depletion gave rise to DNA bridges, micronuclei and the activation of STING-Tbk1 signaling, as seen via their phosphorylation (Fig. 3a–d). Inhibition of Tbk1 or the deletion of *cGAS* via Crispr-Cas9 editing abolished the *ISG15* expression and the ISGylation seen upon Nbs1 depletion, as it does for Camptothecin (CPT) or Aphidicolin (Aph) induced ISGylation (Fig. 3e–g). These data indicate that extranuclear DNA caused by DNA replication stress leads to induction of *ISG15* via the cGAS-STING-Tbk1 pathway in Mre11 complex deficient cells.

### ISGylation impacts the replication fork

**Fig. 4:**
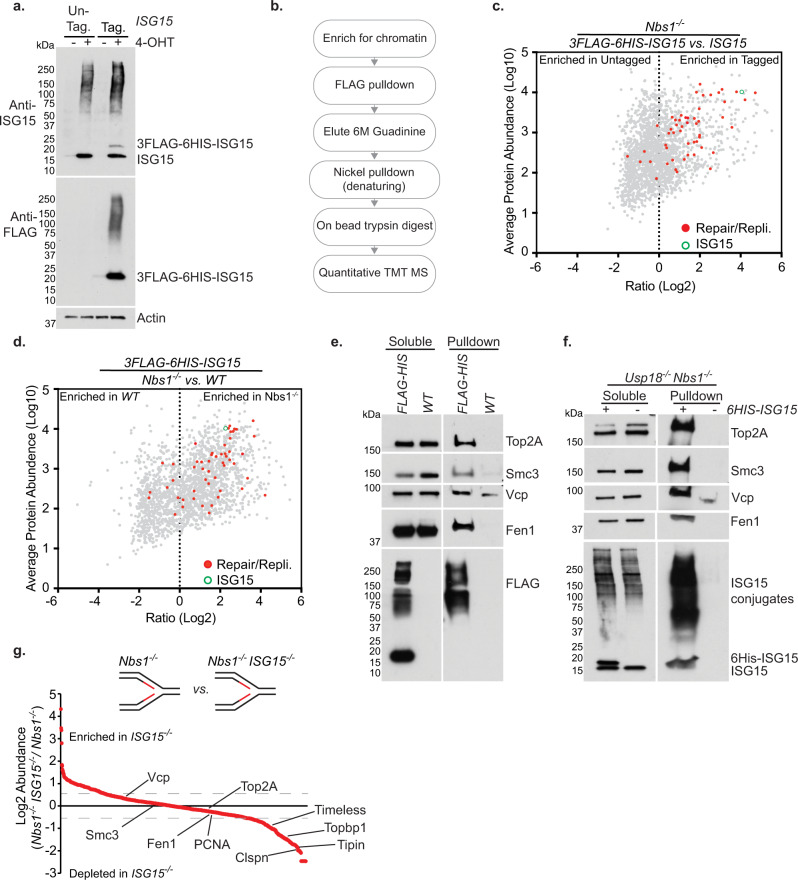
Protein ISGylation occurs at replication forks. **a** Western blot analysis of whole cell extracts taken from *Nbs1*^*f/f*^ and *Nbs1*^*f/f*^ *3FLAG-6HIS-ISG15* with or without 4-OHT induced *Nbs1* deletion. **b** Diagrammatic representation of the approach used to identify ISGylated proteins in **c** and **d**. **c** Mass-spectrometry hits from *Nbs1*^−/−^ *vs.* *Nbs1*^−/−^ *3FLAG-6HIS-ISG15* MEFs. Mean Log2 ratio and Log10 abundance of two independent experiments shown. **d** Mass-spectrometry hits from *3FLAG-6His-ISG15 vs. Nbs1*^*−/−*^ *3FLAg-6HIS-ISG15 MEFs*. Mean Log2 ratio and Log10 abundance of two independent experiments shown. **e** Western blot analysis of nickel pulldowns from the chromatin fraction of *Nbs1*^*−/−*^ and *Nbs1*^*−/−*^ *3FLAG-6HIS-ISG15* MEFs. Soluble inputs and pulldowns from chromatin were extracted from the same cells and run concurrently. **f** Western blot analysis of nickel pulldowns from the chromatin fraction of *Usp18*^*−/−*^ *Nbs1*^*−/−*^ and *Usp18*^*−/−*^ *Nbs1*^*−/−*^ with or without expression of an inducible exogenous 6HIS-ISG15. Soluble and pulldown samples were extracted from the same cells and run concurrently. **g** IPOND-SILAC-MS from *Nbs1*^*−/−*^ *vs. Nbs1*^*−/−*^ *ISG15*^*−/−*^ cells. Proteins identified in at least two out of three independent experiments ranked by mean Log2 ratio. Boundaries for enriched/depleted proteins represented by dotted lines at Log2 abundances of ≥0.6 and ≤ −0.6. Source data are provided with this paper.

Given the enrichment of ISG15 on nascent DNA, and the induction of ISGylation upon replication stress, we reasoned that replication fork associated proteins may be targets of ISGylation. To test that interpretation, *ISG15* was N-terminally tagged with *3FLAG-6HIS* via CRISPR-Cas9 genome editing in *Nbs1*^*f/f*^ MEFs, giving rise to a *3FLAG-6His-ISG15* heterozygote (Fig. 4a). Sequential non-denaturing-FLAG and denaturing-nickel pulldowns were then performed from the chromatin fraction of *Nbs1*^*−/−*^*, Nbs1*^*−/−*^ *3FLAG-6HIS-ISG15* and *3FLAG-6His-ISG15* MEFs, followed by quantitative tandem-mass-tag (TMT)-mass spectrometry (Fig. 4b). This approach avoids the potential caveats associated with *ISG15* over-expression.

We identified ISGylated chromatin associated proteins upon *Nbs1* deletion, 323 of which displayed a twofold or greater enrichment in the 3FLAG-6His-ISG15 *Nbs1*^*−/−*^ sample compared to the (no 3xFLAG-6HIS) *Nbs1*^*−/−*^ control (Fig. 4c and Supplementary Data 3). Approximately 15% of the enriched proteins (49 of the 323 proteins) have known roles in DNA replication and/or repair, implicating ISGylation in the regulation of these pathways (Fig. 4c and Supplementary Data 3). The vast majority of this protein enrichment was dependent on *Nbs1* deletion (Fig. 4d, Supplementary Fig. 5a, Supplementary Data 3). 283 of the 323 proteins seen to be at least 2-fold enriched in the *3FLAG-6His-ISG15 Nbs1*^*−/−*^ cells compared to *Nbs1*^*−/−*^ were also enriched compared to the *3FLAG-6His-ISG15 Nbs1* proficient control (Supplementary Fig. 5a and Supplementary Data 3).

To validate the mass spectrometry results, chromatin was isolated from *Nbs1*^*−/−*^ *3FLAG-6His-ISG15* and *Nbs1*^*−/−*^ cells and denaturing-nickel-pulldowns followed by western blotting performed (Fig. 4e). Four proteins (Top2A, Smc3, Vcp and Fen1) with known roles in DNA replication that showed a similar abundance and log2 ratio to that of ISG15 in the mass spectrometry experiment were subjected to western blotting (Supplementary Data 3 and Fig. 4e). All four proteins, and FLAG, were detected in the samples prepared from the 3FLAG-6HIS-ISG15 cells, with little or no signal present in the samples prepared from the untagged-ISG15 control cells (Fig. 4e).

Further validation was performed using *Nbs1*^*f/f*^ cell lines in which the de-ISGylase, Usp18, was disrupted via CRISPR-Cas9 editing and an exogenous doxycycline inducible 6HIS-ISG15 integrated via lenti-viral infection (*Usp18*^*−/−*^ *+6his-ISG15*). 6His-ISG15 was then isolated under denaturing conditions from chromatin upon *Nbs1* deletion via nickel pulldown (Fig. 4f). All four of the confirmed mass-spectrometry hits, and ISG15, were present exclusively in the samples from the 6His-ISG15 expressing cells (Fig. 4f). The electrophoretic mobility of those four chromatin bound proteins was reduced relative to the soluble fractions, consistent with their being ISGylated (Fig. 4f). Given all four of the proteins tested were confirmed as targets of ISGylation, it is likely that many of the mass spectrometric hits are genuine targets of ISGylation (Fig. 4c, d and Supplementary Data 3).

iPOND followed by Western blotting of the endogenous *3FLAG-6HIS-ISG15 Nbs1*^*f/f*^ cell line showed conjugated, but little free, ISG15 is present on the nascent DNA before Nbs1 depletion and to a markedly greater extent upon Nbs1 depletion. This confirms that ISGylated proteins are associated with nascent DNA and increased upon DNA replication stress (Supplementary Fig. 5b).

ISGylation of proteins associated with nascent DNA could be a nonspecific outcome of cytoplasmic DNA induced interferon signaling. In this scenario, ISGylation at the replication fork would have little biological significance and would simply represent one of myriad sites that get ISGylated when cGAS-STING signaling is activated. Alternatively, ISGylation of nascent DNA associated proteins could be induced by DNA replication stress to stabilize the fork. In this scenario, ISGylation at the fork could serve two nonexclusive functions: to modulate the activities of fork associated proteins, and to promote recruitment or retention of factors that function at the fork.

To address whether ISGylation at the replication fork is functionally significant, *ISG15*^*−/−*^ cells were generated via CRISPR-Cas9 editing in *Nbs1*^*f/f*^ MEFs (*ISG15*^*−/−*^ *Nbs1*^*f/f*^*)* (Supplementary Fig. 5c). iPOND SILAC-Mass-spec analysis was performed three days post 4-OHT addition in *Nbs1*^*f/f*^ *ISG15*^*−/−*^ *vs Nbs1*^*f/f*^ MEFs (Fig. 4g). Comparable enrichments of the MCM helicase components was again observed (Supplementary Fig. 5d). *ISG15*^*−/−*^ *Nbs1*^*−/−*^ cells showed both enrichment and depletion of proteins on nascent DNA compared to *Nbs1*^*−/−*^ cells, with 162 proteins being enriched (log2 ratio ≥0.6) and 213 proteins depleted (log2 ratio ≤ −0.6) (Fig. 4g and Supplementary Data 4). 54 of the proteins depleted on nascent DNA in the *ISG15*^*−/−*^ *Nbs1*^*−/−*^ cells were proteins with a known role in replication fork stabilization, processing or checkpoint activation. These included the fork stabilization proteins Topbp1, Tipin, Timeless and Claspin (Fig. 4g, Supplementary Fig. 5e and Supplementary Data 4). The abundance of bona fide ISGylation targets Fen1, VCP, Smc3 and Top2A at the replication fork did not change in *ISG15*^*−/−*^ cells, suggesting that their ISGylation does not regulate their abundance or localization, but may promote interactions with other factors that contribute to replication fork stability (Fig. 4g and Supplementary Data 4). Collectively, these data suggest that ISGylation of proteins associated with nascent DNA has a direct impact on the replisome. The data also show that the loss of ISG15 leads to a significant depletion of proteins from the replication fork.

### ISG15 suppresses replication stress and promotes genome stability

**Fig. 5:**
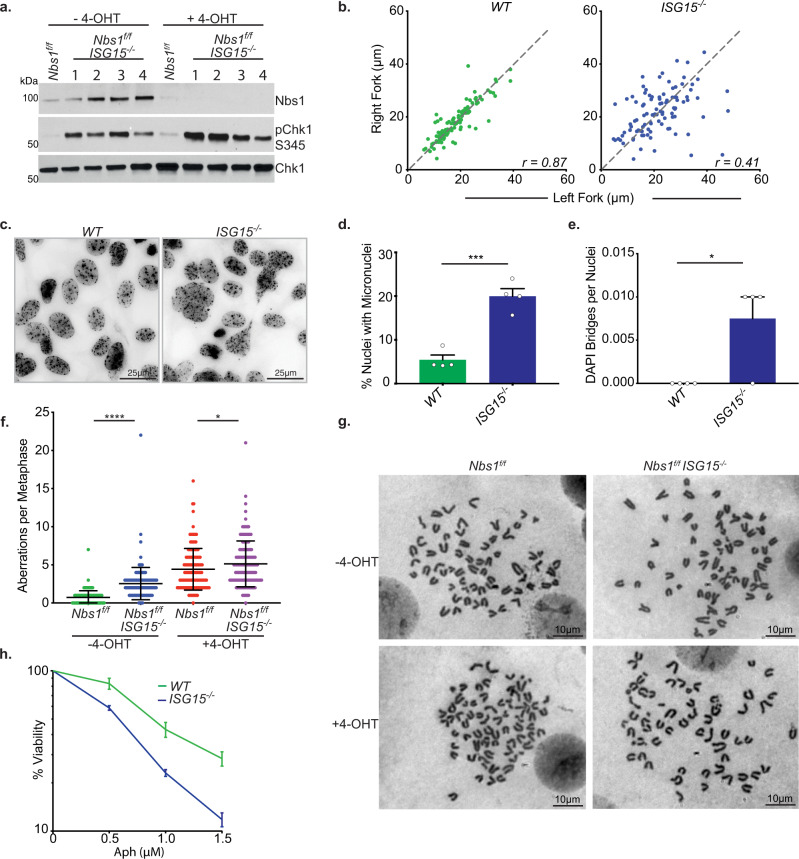
*ISG15*^*−/−*^ MEFs exhibit replication stress and genomic instability. **a** Western blot of whole cell extracts in *Nbs1*^*f/f*^ and *Nbs1*^*f/f*^ *ISG15*^*−/−*^ MEFs with or without *Nbs1* deletion. Lanes labelled 1-4 represent independent *Nbs1*^*f/f*^ *ISG15*^*−/−*^ clones. **b** DNA combing analysis of the indicated cell lines. Left-Right ratios of CldU tracts initiating from the same origin were calculated. Combined data from three independent experiments plotted. *WT* *n* = 107, *ISG15*^*−/−*^ *n* = 99. Pearson’s co-efficient (*r*) shown. **c** Example images for the data shown in d and e. Between 118 and 162 cells were counted per genotype per biological repeat (*WT* *n* = 643, *ISG15*^*−/−*^ *n* = 592). **d** Percentage of nuclei with micronuclei in the indicated cell lines. Mean of four independent experiments with SEM. Two-tailed unpaired *t*-test, *** = *p* 0.0004. **e** Quantification of DAPI stained DNA bridges in the indicated cell lines. Mean of four independent experiments with SEM. Two tailed unpaired *t*-test, * = *p* 0.0240. **f** Quantification of metaphase spreads with or without induction of *Nbs1* deletion. Combined data from three independent experiments. Between 45 and 52 metaphases were counted per condition per biological repeat (*Nbs1*^*f/f*^ −4OHT *n* = 152; *Nbs1*^*f/f*^ *ISG15*^*−/−*^ −4-OHT *n* = 148; *Nbs1*^*f/f*^ + 4-OHT *n* = 144; *Nbs1*^*f/f*^ *ISG15*^*−/−*^ + 4-OHT *n* = 146). Mean with SD shown. Two-tailed Mann–Whitney test ****= *p* < 0.0001, *= *p* 0.0207. **g** Example images for data shown in f. **h** Viability ten days after 16 h aphidicolin (Aph) treatment as seen via colony forming assay. Mean of three independent experiments with SEM. Source data are provided with this paper.

Given the decreased abundance of replication fork stabilizing proteins at ISG15 deficient forks, we hypothesized ISG15 may be required in the suppression of DNA replication stress. Indeed, ISG15 deficient cells exhibited chronic ATR activation as indicated by tonic Chk1 phosphorylation (Fig. 5a). Chk1 phosphorylation was further increased upon *Nbs1* deletion, indicating that ISG15 may be acting to suppress replication stress during unperturbed DNA replication as well as upon *Nbs1* deletion (Fig. 5a).

To directly assess whether ISG15 influences replication fork progression, DNA combing was performed in *ISG15*^*−/−*^ *MEFs*. *ISG15*^*−/−*^ cells did not display a decrease in median replication fork speed (Supplementary Fig. 6a). However, compared to *WT* a greater asymmetry in CldU fiber lengths emanating from the same origin was observed, suggesting replication fork stalling. (Fig. 5b). *ISG15*^*−/−*^ cells exhibited a marked increase in sister CldU tract asymmetry (*WT* *r* = 0.87; *ISG15*^*−/−*^ *r* = 0.41) (Fig. 5b) similar to that seen upon *Nbs1* deletion (Fig. 1c). Consistent with replication fork stalling a 54% reduction in mean EDU intensity could be seen in *ISG15*^*−/−*^ cells compared to *ISG15* proficient cells via flow cytometry (Supplementary Fig. 6b–d). *ISG15*^*−/−*^ cells also exhibited DNA bridges (0.006 per nucleus) and a 20% increase in micronuclei per cell compared to *WT* cells (Fig. 5c–e).

Replication fork stalling in ISG15 deficient MEFs was associated with genomic instability. Metaphase spreads were performed in *Nbs1*^*f/f*^ and *Nbs1*^*f/f*^ *ISG15*^*−/−*^ MEFs with or without 4-OHT addition (Fig. 5f, g). A 3.5-fold increase in aberrations per metaphase can be seen in the *ISG15*^*−/−*^ cells compared to *ISG15* proficient cells (Fig. 5f, g). Consistent with a role for ISG15 in suppressing replication stress, deletion of *Nbs1* in an *ISG15*^−/−^ background led to increased genomic aberrations compared to either deletion alone (Fig. 5f, g). Cells lacking *ISG15* have an increase in all aberration types compared to *WT* cells, with the largest increase being in fusions and DNA fragments, indicative of DNA replication defects, much like that seen in *Nbs1* deficient cells (Supplementary Fig. 6e). Moreover, ISG15 deficiency rendered cells sensitive to aphidicolin, exhibiting a 2.5-fold increase in sensitivity compared to *WT* cells, as assessed by colony formation assays (Fig. 5h and Supplementary 6f). Thus, ISG15 plays a direct role in mitigating replication stress.

Overall, the data obtained in this study reveal a previously unrecognized role for ISG15 at replication forks during unperturbed DNA replication and upon replication stress conditions. This observation reveals a direct link between innate immune signaling and the mechanisms by which cells prevent DNA damage during DNA replication.

## Discussion

Multiple lines of evidence indicate that the Mre11 complex functions in proximity to the replication fork and is integral to the process of DNA replication^9–11,34^. This study was undertaken to examine the effect of acute Mre11 complex inactivation on the constituents and progression of the replication fork. Upon Nbs1 depletion, rapid accumulation of cytoplasmic DNA as well as indices of DNA replication stress, including Chk1 activation, reduced replication fork velocity and replication fork stalling, were detected. In addition, changes in the proteins that associate with chromatin in the proximity to the replication fork was observed.

We previously observed that overexpression of *Chk1* rescues the viability of cells in which the Mre11 complex is destabilized by alterations of the Nbs1-Mre11 interface^35^. These results resonate with that observation and underscore the link between Mre11 complex deficiency and the induction of replication stress, a state that can be mitigated by activation of the ATR-Chk1 axis of the DDR^35^.

Several of the proteins depleted from nascent DNA in Mre11 complex deficient cells play important roles in mitigating replication stress and have been detected at the fork under stressed conditions^25,34^. Their loss at the replication fork may contribute to the induction of DNA replication stress upon acute Nbs1 depletion.

A number of Mre11 complex functions may also underlie the replication stress phenotype associated with acute Mre11 complex inactivation. The complex has been shown to be involved in the removal of R-loops upon oncogene activation, the removal of trapped topoisomerase cleavage complexes, in the recruitment of cohesin to HU stalled forks, and in the processing of secondary DNA structures in replicating chromosomal regions^36–41^.

The primary focus of this study was on one of the proteins most enriched on nascent DNA in response to Nbs1 depletion. ISG15, a ubiquitin like modifier that is induced via cGAS-STING detection of cytoplasmic DNA was enriched by 38-fold in Nbs1 depleted cells (Fig. 1e). Replication stress and the ensuing cytoplasmic DNA induced by either Mre11 complex inactivation or APH and CPT treatment led to induction of *ISG15* expression and ISGylation of proteins on nascent DNA, many of which are involved in DNA replication (Fig. 4c, d).

**Fig. 6:**
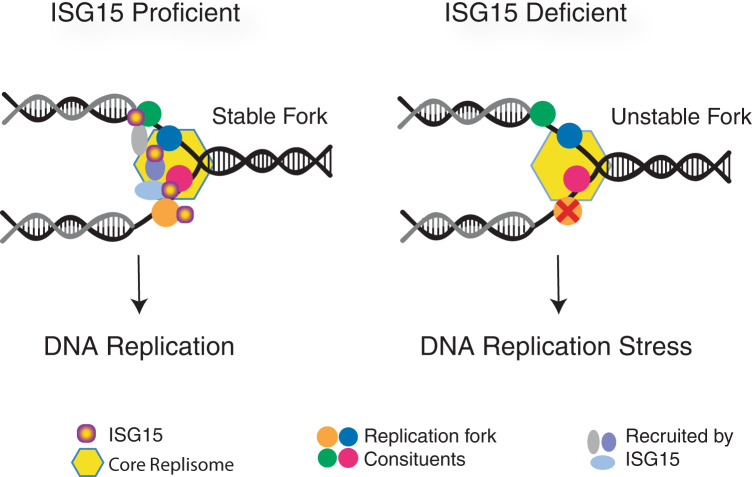
Model. Diagram predicting how ISG15 may be functioning at replication forks to maintain genomic stability. ISG15 proficient cells: ISGylation stabilizes protein interactions and regulates protein functions at stalled replication forks, thus promoting faithful DNA replication. ISG15 deficient cells: No ISGylation of replication fork associated proteins upon fork stalling. Depletion of proteins required for fork stability and/or loss of protein regulation results in replication stress phenotypes. Red cross represents loss of protein activity. See main text for details. Source data are provided with this paper.

ISGylation in response to DNA replication stress appears to influence the recruitment and retention of replication fork proteins. Deletion of ISG15 resulted in changes in protein abundance on nascent DNA, replication stress phenotypes, and genomic instability (Figs. 4g, 5a–h). The data support the idea that ISGylation of replication fork associated proteins is a previously unrecognized output of cGAS signaling that promotes DNA replication under stressed as well as normal conditions (Fig. 6).

Acute Nbs1 depletion, treatment with aphidicolin and treatment with CTP each caused the accumulation of cytoplasmic DNA in the form of DNA bridges and micronuclei (Fig. 3a–c, Supplementary Fig. 4a–c). This, in turn, sustained cGAS-STING-Tbk1 dependent induction of *ISG15* and ISGylation of proteins associated with nascent DNA (Fig. 3d–g and 4c–f).

The cGAS signaling induced by these structures is unlikely to account for the initiation of ISGylation on nascent DNA, as DNA bridges and micronuclei are a downstream consequence of DNA replication stress. Thus, alternative sensors most likely activate cGAS signaling during DNA replication.

Short DNA fragments arising from the resection of broken or reversed replication fork structures are the likely initiators of ISGylation at replication forks. These DNA fragments have been shown to migrate into the cytoplasm and activate cGAS signaling, presumably inducing ISGylation during S-phase^42,43^.

Alternatively, sensing of extrachromosomal nuclear DNA may lead to the ISGylation of replication fork associated proteins. Although a portion of cGAS localizes to the nucleus, chromatin does not activate cGAS-STING-TBk1 signaling^44–46^. However, cGAS could be activated by aberrant DNA structures or non-chromatinized DNA fragments arising at stalled forks.

Other nuclear DNA receptors such as IFI204 and IFI205, which are orthologous to IFI16, a human DNA sensor in the interferon signaling pathway could also initiate the signaling that induces ISGylation at the fork^47,48^. Notably, both IFI204 and IFI205 were enriched at the replication fork upon *Nbs1* deletion (Fig. 1e and Supplementary Data 2). In support of this possibility, IFI16 induces cGAS independent, but STING dependent gene expression in response to etoposide treatment^49^.

To date three ISG15 E3 ligase have been identified, HERC6 (HERC5 in human), Trim25 (EFP) and ARIH1 (HHARI)^50^. HERC6 functions solely as an ISG15 E3 ligase and is required for the majority of ISGylation occurring in the cell. Whereas Trim25 and ARI1 also function as Ubiquitin E3s. The ability of E3 ligases to catalyze both Ubiquitination and ISGylation raises the possibility that more of the 600-700 predicted Ubiquitin E3s^51^ could also be functioning in ISGylation.

We did not identify any of the three known ISG15 E3s upon Nbs1 depletion in our iPOND data sets (Supplementary Data 1, 2, 4). However, a comprehensive iPOND-SILAC study across four human cell lines identified both Trim25 and ARIH1 on both nascent DNA, as well as mature chromatin, giving the possibility that they may be contributing to the ISGylation observed at replication forks^12^. Trim25 has also been shown to localize to nascent DNA via iPOND followed by western blotting in mouse embryonic stem cells (mESCs) and, to a lesser extent, in NIH3T3 cells^52^. Knockdown of Trim25 leads to a defect in replication fork restart upon HU treatment, that cannot be rescued via the expression of a *Δ-ring-Trim25* mutant^52^. However, current data suggests this is due to loss of Ubiquitin E3 ligase activity^52^. Thus, further study is required to identify the ISG15 E3 ligase responsible for the ISGylation occurring at replication forks.

Nbs1 inactivation led to roughly equal numbers of proteins enriched and depleted at the replication fork (Fig. 1e and Supplementary Data 2). In contrast, fifty more replication fork proteins were depleted in ISG15 deficient *Nbs1*^*−/−*^ cells than enriched (Fig. 4g and Supplementary Data 4). These data suggest that ISGylation may differentially influence the recruitment or retention of factors at the fork.

However, ISG15 deficiency did not affect the abundance of its bona fide target proteins (Top2A, Smc3, Vcp and Fen1) at the replication fork (Fig. 4e–g and Supplementary Data 3–4). Thus, ISGylation does not appear to influence the localization or abundance of its target proteins on nascent DNA. Rather, we propose that ISGylation influences recruitment or retention of other proteins via modulating non-covalent protein-protein interactions, as has been proposed for sumoylation of proteins at sites of DNA damage^53–55^ (Fig. 6). For example, the Smc3 interacting proteins Esco2, Nipbl and Wapal; the Vcp interacting proteins Ufd1l and Nploc4; and the Top2A interacting protein BLM were depleted at the fork in *ISG15*^*−/−*^ MEFs following *Nbs1* ablation (Supplementary Fig. 5e, Supplementary Data 4). Modest reductions of Sumo1 and Sumo2 (log2 ratios of −0.66 and −0.78 respectively) were also seen on nascent DNA (Supplementary Fig. 5e and Supplementary Data 4). Suggesting loss of ISGylation may also impact the regulation of other post translational modifications that occur at replication forks, thus contributing to the changes in protein abundance seen.

We hypothesize that ISG15 mediated recruitment of factors that mitigate replication stress at least partially accounts for Chk1 phosphorylation and asymmetric sister forks in ISG15 deficient cells (Fig. 5a, b). Indeed, the levels of 54 proteins involved in DNA replication/repair including fork stabilization proteins such as Topbp1, Claspin, Timeless and Tipin were reduced on nascent DNA in *ISG15*^*−/−*^ cells upon *Nbs1* deletion (Fig. 4g, Supplementary Fig. 5e and Supplementary Data 4). The deficiency of these proteins could also explain the chromosome aberrations in *ISG15*^*−/−*^ *WT* and *Nbs1*^*−/−*^ cells (Fig. 5f, g, Supplementary Fig. 6e).

These data uncover a novel role for innate immune signaling and ISG15 during DNA replication, and support a model wherein innate immune signaling maintains replication fork stability via the ISGylation of fork associated proteins. ISGylation may in turn stabilize the replisome by regulating protein interactions and functions at the replication fork, and thereby reduce replication stress induced genomic aberrations (Fig. 6).

This proposed role for ISG15 at replication forks is distinct from previous studies implicating ISG15 in DNA replication related processes. ISGylation of PCNA has been shown to occur upon UV damage. In this instance ISG15 functions to terminate translesion synthesis via promoting the re-engagement of the replicative polymerase to PCNA^56^. Our data do not support a similar role for ISGylation of PCNA upon replication fork stalling, nor is there evidence that the Mre11 complex function is linked to translesion synthesis.

Over expression of *ISG15* in unperturbed cells has been reported to non-covalently associate with Recq1. This interaction activates Recq1’s replication restart activity, leading to increased replication fork velocity and subsequent DNA replication stress^57^. In contrast, the effects of ISG15 in this study are mainly due to conjugation to its targets. Induction of *ISG15* under DNA replication stress conditions, such as upon Nbs1 depletion or treatment with CPT or APH, promotes genome stability through conjugation to proteins associated with nascent DNA. Although the effect of *ISG15* overexpression on replication fork velocity is not due to covalent attachments, the data collectively point to the importance of tight regulation of *ISG15* levels for maintaining genome integrity during DNA replication.

The evolutionary basis for the involvement of innate immune signaling in promoting genomic stability during DNA replication is unclear. Several components of the DDR are targeted for destruction or inactivation by virally encoded proteins^58,59^. ISGylation of replication fork associated proteins may serve as a buffer to counteract the otherwise deleterious effects ensuing from DDR inhibition.

Alternatively, given that impediments to DNA replication fork progression are common and often result in the appearance of extrachromosomal DNA (either cyto or nucleoplasmic), DNA sensors from innate immune pathways and their downstream outputs may have become co-opted by the DDR to effect DNA replication quality control in non-infected cells. Further study of ISG15 targets at the replication fork will shed important light on this issue. In the meantime, these findings broaden understanding of the biological impact of interferon signaling, and provide new insight regarding the role of ISGylation in promoting genomic integrity.

## Methods

### Cell lines

SV40 and *p53*^*−/−*^ immortalized MEFs were cultured in DMEM 10% cosmic calf serum (CCS) supplemented with GlutaMAX. SV40 *Nbs1*^*f/f*^ *Cre*^*ERT*^, *Nbs1*^*-/f*^ *Cre*^*ERT*^, *Mre11*^*-/f*^ *Cre*^*ERT*^ and *Atm*^*−/−*^ MEFs were previously published^21,22,60^. For *Nbs1*^*-/f*^ *Cre*^*ERT*^ and *Mre11*^*-/f*^ *Cre*^*ERT*^ *exon 6 or exon 5* respectively were deleted via addition of 250 nM of 4-OHT (Tocris Bioscience, 34-1210) for 48 h (or 24 h for iPOND experiments). The 4-OHT was then removed, and cells harvested at the indicated time points post 4-OHT addition, or at three days post addition if not stated.

CRISPR-CAS9 edited cell lines were constructed using an RNP method. 10 μg of guide RNA (IDT Alt-R CRISPR-Cas9 sgRNA) was combined with 10 μg Cas9 protein (TrueCut V2, Invitrogen, A36498) and incubated at room temperature for 20 min before being nucleofected (Nucleofector II, Amaxa) into 700,000 cells according to manufactures guidelines. A second round of nucleofection was performed 48–72 h later. Single clones were isolated in 96 well plates via limiting dilution or FACs. Clones were genotyped via PCR followed by restriction digest and agarose gel electrophoresis, via sequencing, via ICE analysis (www.synthego.com) and via western blotting. All guide RNAs and genotyping primers were designed using CHOPCHOP (https://chopchop.cbu.uib.no) or Benching software (https://www.benchling.com). Guides, templates, genotyping primers and restriction enzymes are listed in Supplementary Data 5.

For stable expression of Cre^ERT^, *P53*^*−/−*^ or *WT* MEFs were infected with MSCV *Cre*^*ERT2*^ *Puro* (Addgene plasmid #22776) or MSCV *Cre*^*ERT2*^ *Neo* and single clones isolated. For Dox inducible *6HIS-ISG15* MEFs, 6HIS-ISG15 was cloned as a HindIII-NotI fragment from pcDNA3.1 + N-6HIS-ISG15 (Genescript OMu19630) into the PLNCX2 retroviral vector. Retrovirus infected clones stably expressing 6HIS-ISG15 were Isolated.

### Whole cell extract western blots

Whole cell extracts and western blotting was carried out using standard methods. Briefly, total Cell extracts were prepared in 2x Laemmli sample buffer (4% SDS, 20% Glycerol, 120 mM Tris-HCl pH 6.8, 1x beta-mercaptoethanol, 0.002% bromophenol blue). 30–40 μg of extract was loaded into gradient SDS-PAGE gels (Bio-Rad) and transferred to nitrocellulose membranes (Amersham Proton 0.2 μM NC #10600006) blocked in 3–5% skimmed milk TBST and probed with the antibodies listed in Supplementary Data 5. Membranes were developed using either ECL or ECL Prime (Amersham RPN2106/RPN2232) and exposed to Hyblot-ES Autoradiography Film (Denville, #1156p38). Independent *Nbs1*^*f/f*^ *ISG15*^*−/−*^ clones were used for each repeat.

### DNA combing

DNA combing was performed using the Molecular Combing System (Genomic Vision) following their recommended protocol. Briefly, cells were labeled with 100 μM IdU (Millipore Sigma, I-7125) for 30 minutes, washed in warm media, and pulsed for 30-min in 100 μM CldU (Millipore Sigma, C-6891). DNA plugs were then prepared using the FiberPrep DNA extraction Kit (Genomic Vision) from 100,000 cells according to manufactures instructions. Subsequently DNA was combed onto Combicoverslips (Genomic Vision) using the Molecular combing system-MCS (Genomic Vision) at a fixed speed of 300 μm/s to give a stretching factor of 2 kb/μm. Coverslips were dried at 65 °C for 2 h, denatured in 0.5 N NaOH +1 M NaCl for 8 min, and then dehydrated in an ethanol series for 5 min each (70%, 90%,100%) according to the Genomic Vision protocol. They were subsequently blocked in 5% FBS PBS for 1 h at room temperature. Slides were then incubated with anti-CldU (1:10, Abcam #6326) and anti-IdU (1:5, BD Biosciences #347580) in 5% FBS PBS at 4 °C O/N. Slides were washed 3X with 3% FBS PBS and then incubated with the secondary antibodies Goat anti-mouse Alexafluor 488 (Life Technologies, A32723) and Goat anti-Rat Alexafluor 568 (Life Technologies, A11077). Slides were washed 3 times in 5% FBS PBS, and were incubated with anti-ssDNA (Mouse, Millipore MAB3034 clone 16–19) in 3% FBS PBS for 30 min at 37 °C. Following three washes in 5% FBS PBS slides were incubated with Goat anti-mouse Alexafluor 647 (Life Technologies, A21236) in 3% FBS PBS. They were then washed 3X PBS and mounted using Fluoromount-G (Southern Biotech, 0100-01). Slides were imaged using Deltavision Imaging Elite System (GE healthcare) with a CMOS camera on an Olympus IX-71 microscope using 60x objective. CldU tracts were measured using Fiji Software (Version 2.0.0). For fork velocity more than 200 intact fibers (as determined via ss-DNA staining) were counted per sample and more than 80 fibers counted for fork Symmetry. Data is a combination of three independent repeats. Independent *Nbs1*^*f/f*^ *ISG15*^*−/−*^ clones were used for each repeat.

### iPOND

iPOND was performed as previously described^25,26^. Briefly, 400 million cells were grown either in heavy (L-ARG:HCL 13C6, 15N4; L-LYS:2HCL 13C6, 15N2 (Cambridge Isotopes #CNLM-539-H-0.25 and #CNLM-291-H-0.25)) or light (light ARG and LYS equivalents) DMEM 10% Dialyzed FBS (Gibco, #26400-044) supplemented with 100 mg/L proline (Thermo Fisher #88430). Cell were pulsed for 10 min with 10 μM EdU, or for pulse-chase experiments, pulsed for 10 min with 10 μM EdU, washed once with media and incubated with media containing 10 μM thymidine for the indicated times. Cells were then fixed for 10 min in 1% Formaldehyde, quenched for 15 min via the addition of glycine, and washed 3x in PBS. Cells were harvested, heavy and light cells combined 1:1, and incubated in permeabilization buffer (0.25% Triton x100, PBS) for 30 min at room temperature. Cells were then washed and incubated in Click reaction buffer (10 μM Biotin azide, 10 mM sodium ascorbate, 2 mM CuSO_4_) for 2 h. Following washing, cells were lysed in 1% SDS, 50 mM Tris pH 8 + protease inhibitors and sonicated at 35% amplitude for 26 s on 40 s off with a Branson 450 Digital Sonifier with micro tip. Lysates were cleared via centrifugation and filtration through a 80 μM nylon membrane (Millipore #NY8004700), then diluted 1:1 with PBS. They were subsequently incubated for 1 h at room temperature with MyOne Streptavidin C1 Dynabeads (Invitrogen #65001). Beads were washed sequentially with lysis buffer, low salt wash buffer (1% Triton x-100, 20 mM Tris pH 8, 2 mM EDTA, 150 mM NaCl), high salt wash buffer (1% Triton x-100, 20 mM Tris pH 8, 2 mM EDTA, 500 mM NaCl) and lithium chloride buffer (100 mM Tris pH8, 500 mM LiCl, 1% Igepal). Proteins were eluted in 2x Laemmli buffer at 95 °C for 45 min. Samples were then run 0.5 cm into a gradient SDS-PAGE gel for mass spectrometry (see below). Independent *Nbs1*^*f/f*^ *ISG15*^*−/−*^ clones were used for each repeat. Venn Diagrams were created on https://bioinformatics.psb.ugent.be/webtools/Venn/.

### EDU flow cytometry

Cells were pulsed for 30 min with 15 μM EDU and samples prepared using the Click-iT Plus EdU Alexa Fluor 647 Flow Cytometry Assay Kit (Thermo Fisher #C10634) according to manufactures instructions. Total DNA was stained with FxCycle Violet Stain (DAPI) (Thermo Fisher Scientific, F10347). Cells were analyzed on a Fortessa 3 flow cytometer (BD). Doublets were gated out and 10,000 cells analyzed. The % of EDU positive cells, average EDU intensity and cell cycle distribution calculated using FlowJo software.

### Cellular fractionation

Cells were harvested into Buffer A (10 mM Hepes pH7.9, 10 mM KCl, 1.5 mM MgCl_2_, 0.34 M sucrose, 10% Glycerol, 1 mM DTT, 10 mM NaF, 1 mM Na_2_VO_3_, 1 mM PMSF, 1 mM Benzamidine, Complete EDTA-Free Protease Inhibitor Cocktail (Roche)), lysed via addition of Triton X-100 (final concentration 0.2%) and incubated for 15 min at 4 °C. Cell lysates were centrifuged at 1500 × *g* for 2 min. The supernatant contains the soluble fraction. The nuclei pellet was washed twice with Buffer A and resuspended in Buffer B (10 mM Hepes pH7.9, 3 mM EDTA, 2 mM EGTA, 1 mM DTT, 10 mM NaF, 1 mM Na_2_VO_3_, 1 mM PMSF, 1 mM Benzamidine, Complete EDTA-Free Protease Inhibitor Cocktail (Roche)). Samples were incubated at 4 °C for 10 min and centrifuged at 2000 × *g* for 2 min. The chromatin pellet was washed 3× in Buffer B and resuspended in Laemmli buffer and boiled for 5 min.

### RT-qPCR

Total RNA was extracted from *Nbs1*^*f/f*^ *Cre*^*ERT*^ cell lines 3 days post 4-OHT addition or without 4-OHT addition using the RNeasy Mini Kit (Qiagen) with DNase according to manufactures instructions. cDNA was synthesized using RNA to cDNA EcoDry Premix Double Primed (Takara Biosciences) according to manufacturer’s instructions. qPCR was performed using SsoAdvanced Universal SYBR Green Supermix (Biorad #1725272) on a CFX384 real time system (BioRad). Target gene expression was normalized to *GAPDH*, and relative expression determined with comparative CT method. Primers used are listed in Supplementary Data 5.

### DNA Bridges and Micronuclei staining

Cells grown on coverslips were fixed with 4% (v/v) formaldehyde in PBS for 20 min at RT then permeabilized in ice cold methanol for 15 min at room temperature. Cells were then incubated with 1 μg/ml DAPI in 1% BSA PBST for 1 h at room temperature, washed 3× in PBST and mounted using Fluoromount-G (Southern Biotech, 0100-01). Slides were imaged using Deltavision Imaging Elite System (GE healthcare) with a CMOS camera on an Olympus IX-71 microscope using 60× objective. The images analyzed using Fiji software. Independent *Nbs1*^*f/f*^ *ISG15*^*−/−*^ clones were used for each repeat.

### Pull-downs

For 3FLAG-6HIS-ISG15 pulldown and mass spectrometry chromatin was isolated as described above from 8 × 15 cm plates per sample (~100 million cells). The chromatin was resuspended in IP buffer (20 mM Tris pH 7.5, 300 mM NaCl, 10 mM MgCl_2_, 0.5% Triton X-100, 1 mM AEBSF, 1 mM Benzamidine, Complete EDTA-Free Protease Inhibitor Cocktail (Roche)) and sonicated 3× 25 s on, 40 s off at 35% amplitude on ice using a Branson 450 Digital Sonifier with a micro tip. Benzonase (Millipore, #70746-4) was added, and samples incubated on Ice before being centrifuged at 16,000 × *g* for 10 min at 4 °C. Supernatant was added to 50 μl Packed volume of Anti-FLAG M2 Agarose Affinity Resin (Sigma, F1804) and samples rotated O/N at 4 °C. Resin was washed 3X in IP buffer and proteins eluted in denaturing buffer (6 M Guanidine-HCl, 10 mM Tris, sodium phosphate buffer pH 8) containing 5 mM beta mercaptoethanol, 5 mM imidazole for 45 min at room temperature whilst shaking. Supernatant was added to 50 μl of Ni-NTA Agarose beads (Qiagen, # 1018244) and rotated at 4 °C O/N. Beads were washed 1x in denaturing buffer containing 5 mM beta-mercaptoethanol, 0.1% Triton X-100; 1× in pH 8 urea wash buffer (8 M Urea, 10 mM Tris, 0.1% triton, 100 mM sodium phosphate buffer pH 8, 5 mM beta-mercaptoethanol) and 3× in pH 6.3 urea wash buffer (8 M Urea, 10 mM Tris, 0.1% Triton X100, 100 mM sodium phosphate buffer pH 6.3, 5 mM beta-mercaptoethanol). For mass-spectrometry beads were resuspended in 10 mM Epps buffer and transferred to Ultrafree-MC-HV-centrifugal Filter—Durapore-PDVF 0.45 μM columns. They were then washed 3× in 10 mM Epps removed from the columns and analyzed via on bead trypsin digest and TMT-mass spectrometry (See below).

For western blotting chromatin was isolated as described above from 8 × 15 cm plates. It was then resuspended in the denaturing buffer and nickel pulldowns were performed as above with the following exceptions. After the final Urea pH 6.3 wash, beads were washed 1× in 10 mM Epps without transfer to the PDVF column. The proteins were eluted from the nickel beads in 200 mM Imidazole, 5% SDS, 150 mM Tris-HCl pH 6.7, 30% glycerol, 720 mM beta-mercaptoethanol, 0.0025% bromophenol blue for 40 min shaking at room temperature followed by 1–2 min at 95 °C. All sample was loaded on a gradient SDS-PAGE gel (Biorad) for western blotting as described above. This protocol was modified from^61^.

The same protocol was followed for 6HIS-ISG15 pulldowns and western blotting, with the addition of a 24 h 1 μM doxycycline treatment before harvesting the cells. This protocol was modified from^61^.

### Metaphase spreads

Cells were treated with 100 ng/ml of KaryoMAX colcemid (Life technologies) for 1 h and harvested. Cells were swelled in 0.075 M KCl for 15 min at 37 °C and fixed in ice-cold 3:1(v/v) methanol: acetic acid at −20 °C O/N. Samples were dropped on slides and stained with 5% Giemsa (Sigma) at room temperature for 5 min. Following 3× washes in dH2O slides were dried for several hours at room temperature and mounted with Permount medium (Fisher Scientific). Metaphases were imaged on Olympus IX50 microscope with an Infinity 3 camera (Lumenera) using a 100X Objective. More than 40 spreads were analyzed per sample per repeat. Independent *Nbs1*^*f/f*^ *ISG15*^*−/−*^ clones were used for each repeat.

### Colony forming assay

500–1500 cells were plated on 10 cm plates in triplicate. 6 h later cells were treated with the indicated doses of Aphidicolin (Calbiochem, CAS 38966-21-1) for 16 h. Cells were grown for eight to 10 days and colonies stained with crystal violet (0.5% crystal violet, 25% methanol). The colonies were counted for each technical repeat and the average calculated. Three independent experiments were performed using independent *Nbs1*^*f/f*^ *ISG15*^*−/−*^ clones.

### Mass spectrometry

For iPOND-SILAC gel sections were excised, washed, reduced with DTT, alkylated with IAA, and digested with trypsin (or Lys-C for Fig. 4g) overnight at 37 °C. Peptides were desalted with homemade C18 StageTips^62^, then vacuum centrifuged by SpeedVac. Peptides were reconstituted in 0.1% formic acid (FA) and analyzed by LC-MS/MS using a NanoAcquity (Waters) with a 100 μm inner-diameter × 10 cm- length C18 column (1.7 μm BEH130, Waters) configured with a 180 μm × 2 cm trap column coupled to a Q-Exactive Plus mass spectrometer (Thermo Fisher Scientific). Peptides were eluted over a linear gradient of 0–30% acetonitrile (0.1% FA) in water (0.1% FA) over 150 or 200 min at 300 nL/min. The QE Plus was operated in automatic, data-dependent MS/MS acquisition mode with one MS full scan (380–1800 m/z) at 70,000 mass resolution and up to ten concurrent MS/MS scans for the ten most intense peaks selected from each survey scan. Survey scans were acquired in profile mode and MS/MS scans were acquired in centroid mode at 17,500 resolution and isolation window of 1.5 amu and normalized collision energy of 27. AGC was set to 3 × 10^6^ for MS1 and 1 × 10^3^ and 60 ms IT for MS2. Charge exclusion of unassigned and greater than 6 enabled with dynamic exclusion of 15 s.

All MS/MS data was processed with the MaxQuant software (Max Planck Institute of Biochemistry, Martinsried, Germany; version 1.5.3.30). The default was used for first search tolerance and main search tolerance: 20 and 6 ppm, respectively. Labels were set to Arg10 and Lys8. MaxQuant was set up to search the reference mouse proteome database downloaded from Uniprot on Aug 1st, 2017. Maxquant performed the search assuming trypsin digestion with up to two missed cleavages. Peptide, site, and protein FDR were all set to 1% with a minimum of 1 peptide needed for identification but two peptides needed to calculate a protein level ratio. The following modifications were used as variable modifications for identifications and included for protein quantification: oxidation of methionine, acetylation of the protein N-terminus, ubiquitination of lysine, phosphorylation of serine, threonine and tyrosine residues, and carbamidomethyl on cystine. *P*-values were generated from normalized log2 intensities using 2-tailed test. GO terms were generated using the Mouse Genome Informatics web site (http://www.informatics.jax.org).

For 3FLAG-6HIS-ISG15 pulldown TMT mass spectrometry nickel Beads were transferred to Eppendorf tubes with 500 μL of 25 mM EPPS (pH 8.5), centrifuged at 500 × g and the supernatant removed. 70 μL of 50 mM EPPS was added to the beads and transferred to new tubes. Trypsin and Lys-C (600 ng each) was used for on-bead digest overnight at 37 °C. Fresh 0.45 μm filters (Millipore Ultrafree-MC-HV; UFC30HV25) were preconditioned with two 300 μL washes of 50 mM EPPS. Beads were transferred onto the preconditioned filters and centrifuged at 8000 × *g* for 3 min to isolate peptides. The flow through (peptides) were transferred to new Eppendorf tubes and anhydrous acetonitrile (ACN) was added to make a 30% ACN solution. TMT-tags (11plex; 10 μL of 20 g/L) were added to each sample and incubated for 1 hr. A ratio check was performed to confirm label efficiency then quenched with hydroxylamine to a final concentration of 0.3% for 15 min. Samples were then pooled and vacuum-centrifuged to dryness. The pooled sample was reconstituted in 300 μL 1% trifluoroacetic acid (TFA), fractionated by Pierce™ High pH Reversed-Phase Peptide Fractionation Kit (Cat. No.: 84868) into 8 fractions, then reconstituted into 0.1% FA for LC-MS/MS analysis.

Fractions were analyzed using a Thermo Easy-nLC 1200 (Thermo Fisher Scientific) with a 50 cm (inner diameter 75 μm) EASY-Spray Column (PepMap RSLC, C18, 2 μm, 100 Å) heated to 60 °C coupled to a Orbitrap Fusion Lumos Tribrid Mass Spectrometer (Thermo Fisher Scientific). Peptides were separated at a flow rate of 300 nL/min using a linear gradient of 1 to 35% acetonitrile (0.1% FA) in water (0.1% FA) over 4 h and analyzed by SPS-MS3. MS1 scans were acquired over a range of m/z 375–1500, 120 K resolution, AGC target of 4e5, and maximum IT of 50 ms. MS2 scans were acquired on MS1 scans of charge 2–7 using an isolation of 0.7 m/z, collision induced dissociation with activation of 35%, turbo scan and max IT of 50 ms. MS3 scans were acquired using specific precursor selection (SPS) of 10 isolation notches, m/z range 100–1000, 50 K resolution AGC target of 1e5, HCD activation of 65%, and max IT of 150 ms. The dynamic exclusion was set at 38 s.

Raw data files were processed using Proteome Discoverer (PD) version 2.4.1.15 (Thermo Scientific). For each of the TMT experiments, raw files from all fractions were merged and searched with the SEQUEST HT search engine with a Mouse UniProt protein database downloaded on 2019/12/13 (92,249 entries). Cysteine carbamidomethylation was specified as fixed modifications, while Methionine oxidation, acetylation of the protein N-terminus, TMT6plex (K) and TMT6plex (N-term), phosphorylation (STY), GGTMT6plex (K) were set as variable modification. The precursor and fragment mass tolerances were 10 ppm and 0.6 Da respectively. A maximum of two trypsin missed cleavages were permitted. Searches used a reversed sequence decoy strategy to control peptide false discovery rate (FDR) and 1% FDR was set as threshold for identification.

### Statistics and reproducibility

Statistical analysis was performed using GraphPad Prism 9.0.0. A two-tailed unpaired *t-* test was used to perform a statistical analysis comparing two samples. Mann-Whitney test was used for fork velocity and metaphase spread analysis. Graphs were created using GraphPad Prism 9.0.0 or Microsoft Excel 16.54. All experiments were repeated at least three times, with the exception of Fig. 4c–f which were repeated twice, giving similar results. For *ISG15*^*−/−*^ experiments each biological repeat of an experiment was performed with an independent clone.

### Reporting summary

Further information on research design is available in the Nature Research Reporting Summary linked to this article.

## Supplementary information

Supplementary Information

Description of Additional Supplementary Files

Supplementary Data 1

Supplementary Data 2

Supplementary Data 3

Supplementary Data 4

Supplementary Data 5

Reporting Summary

## Data Availability

Data plotted in the mass-spec graphs, Venn diagrams and data (Figs. 1d, e, 4c, d, g, Supplementary Figs. 2a, b, e, 5a, d, e) are presented in the Supplementary Data. The raw mass spectrometry data generated in this study have been deposited to the ProteomeXchange Consortium via the PRIDE partner repository with the dataset identifier PXD031770. A Source Data file is provided with this paper. Any other data that support this study are available from the corresponding author upon reasonable request.

## Source data

Source Data
