# Supplementary material for: ISG15 conjugation to proteins on nascent DNA mitigates DNA replication stress
Source: Nat Commun. 2022 Oct 10;13:5971. doi: 10.1038/s41467-022-33535-y (PMC9550767; doi:10.1038/s41467-022-33535-y)
Supplement: Supplementary file 1 — Supplementary Information [file 41467_2022_33535_MOESM1_ESM.pdf]

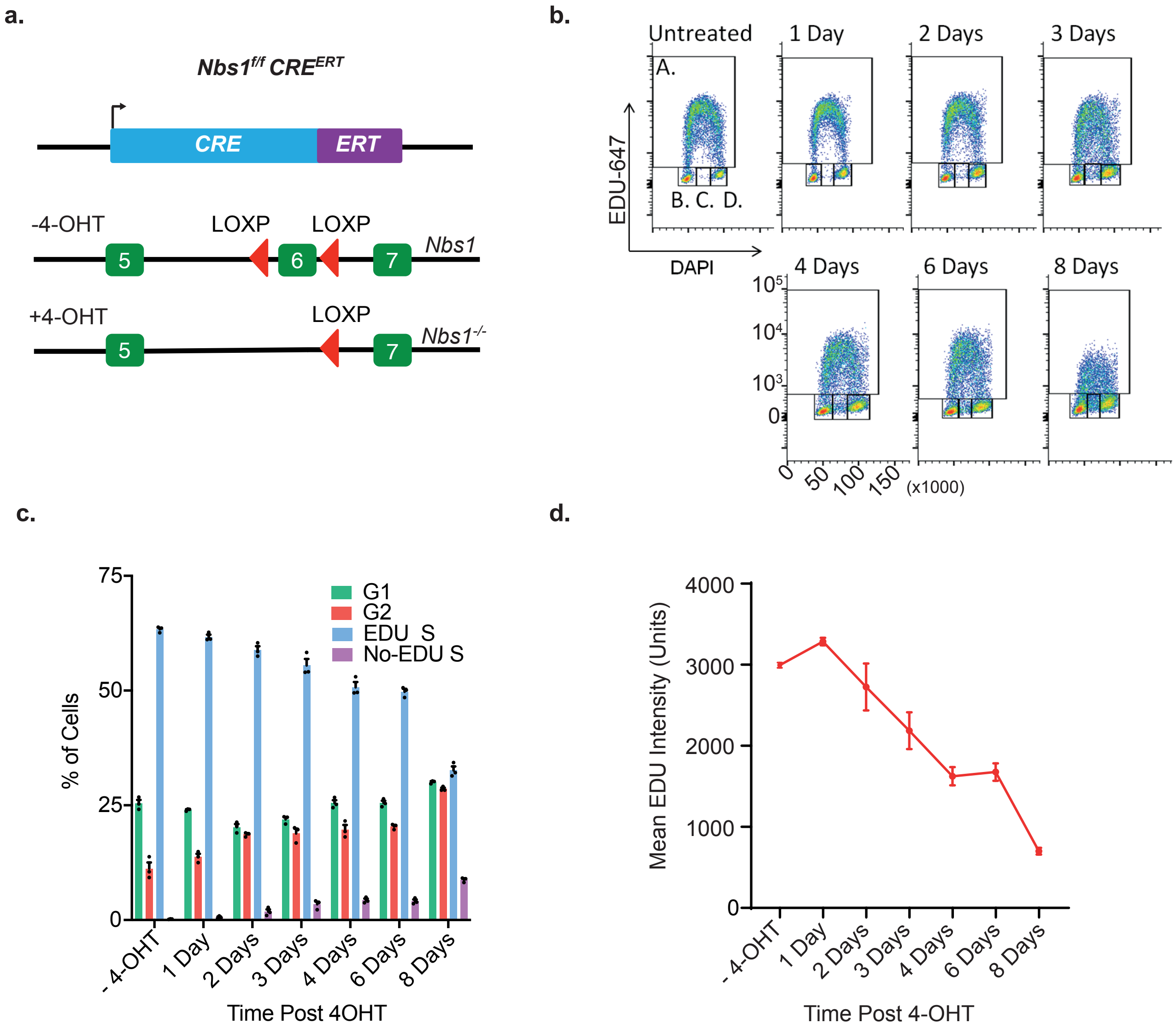

Supplementary Fig. 1. Deletion of *Nbs1* Reduces EDU Incorporation
a. Diagram showing *Nbs1*$^{f/f}$ *Cre*^{ERT} system. **b.** Example EDU flow-cytometry graphs from *Nbs1*$^{f/f}$ cells at indicated days post 4-OHT addition. Box A = EDU positive S-phase cells, box B = G1 Cells, box C = EDU negative S-phase cells, box D = G2 cells. Three independent repeats were performed with similar results. Data are shown in panels c and d. **c.** Quantification of EDU flow-cytometry cell cycle phases. Mean of three independent experiments with SEM. **d.** Quantification of average EDU intensities. Mean of three independent experiments with SEM.

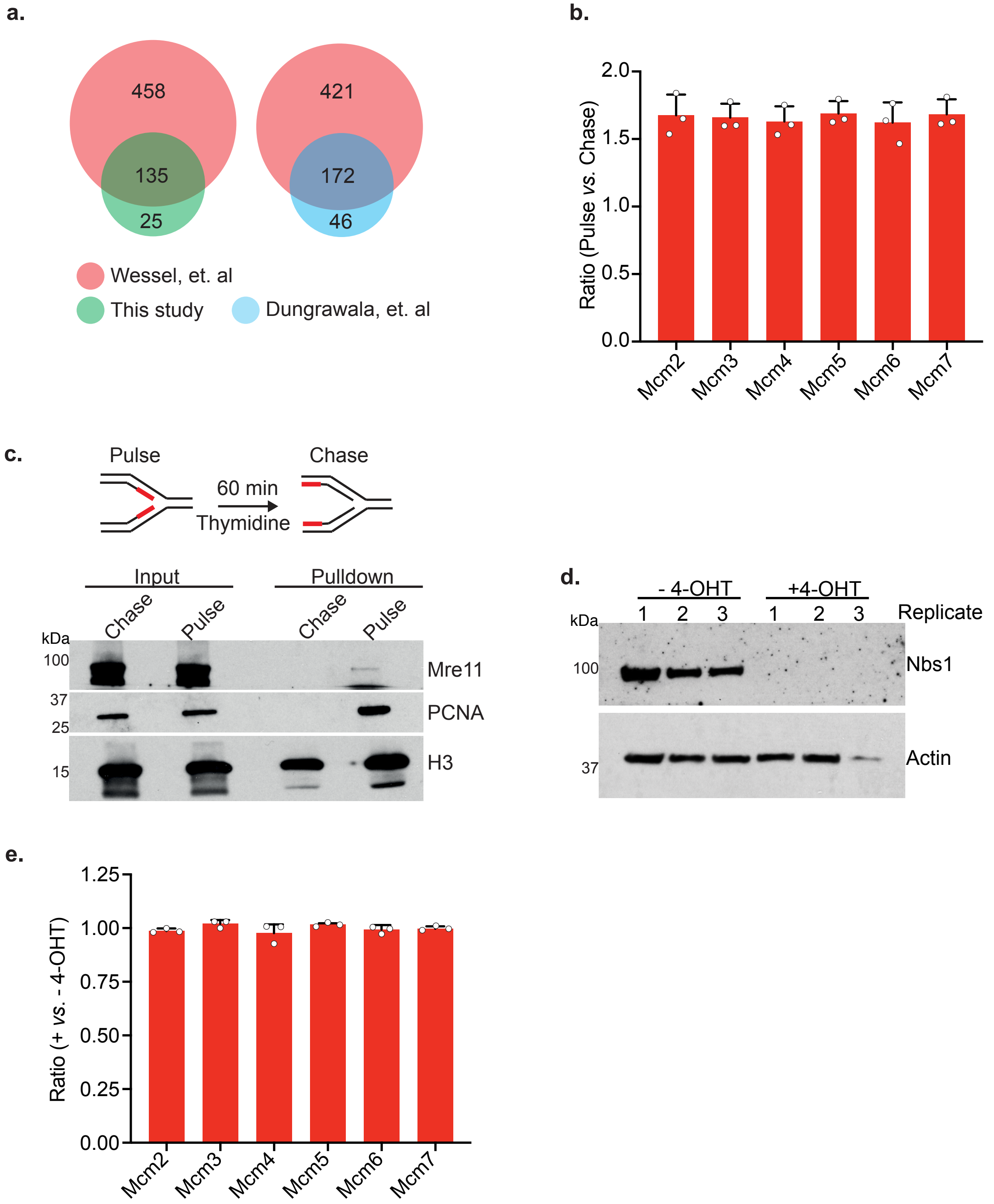

Supplementary Fig. 2. iPOND Controls

a. Venn diagram comparing proteins enriched on nascent DNA between this study and combined data from four human cell lines (Wessel et al. 2019)[12] and between 293 cells (Dungrawala et al. 2015)[25] and Wessel et al. 2019[12]. Associated data shown in Supplementary Table 1. **b.** Mcm control for pulse-chase-SILAC-MS in Fig. 1d. Mean of three independent experiments with SD.**c.** Western blot analysis of iPOND samples taken after a 10 min EDU pulse, with or without a 60 min thymidine chase. **d.** Western blot of whole cell extracts taken immediately before the iPOND experiment shown in Fig. 1e. **e.** Mcm control for the iPOND experiment shown in Fig. 1e. Mean of three independent experiments with SD.

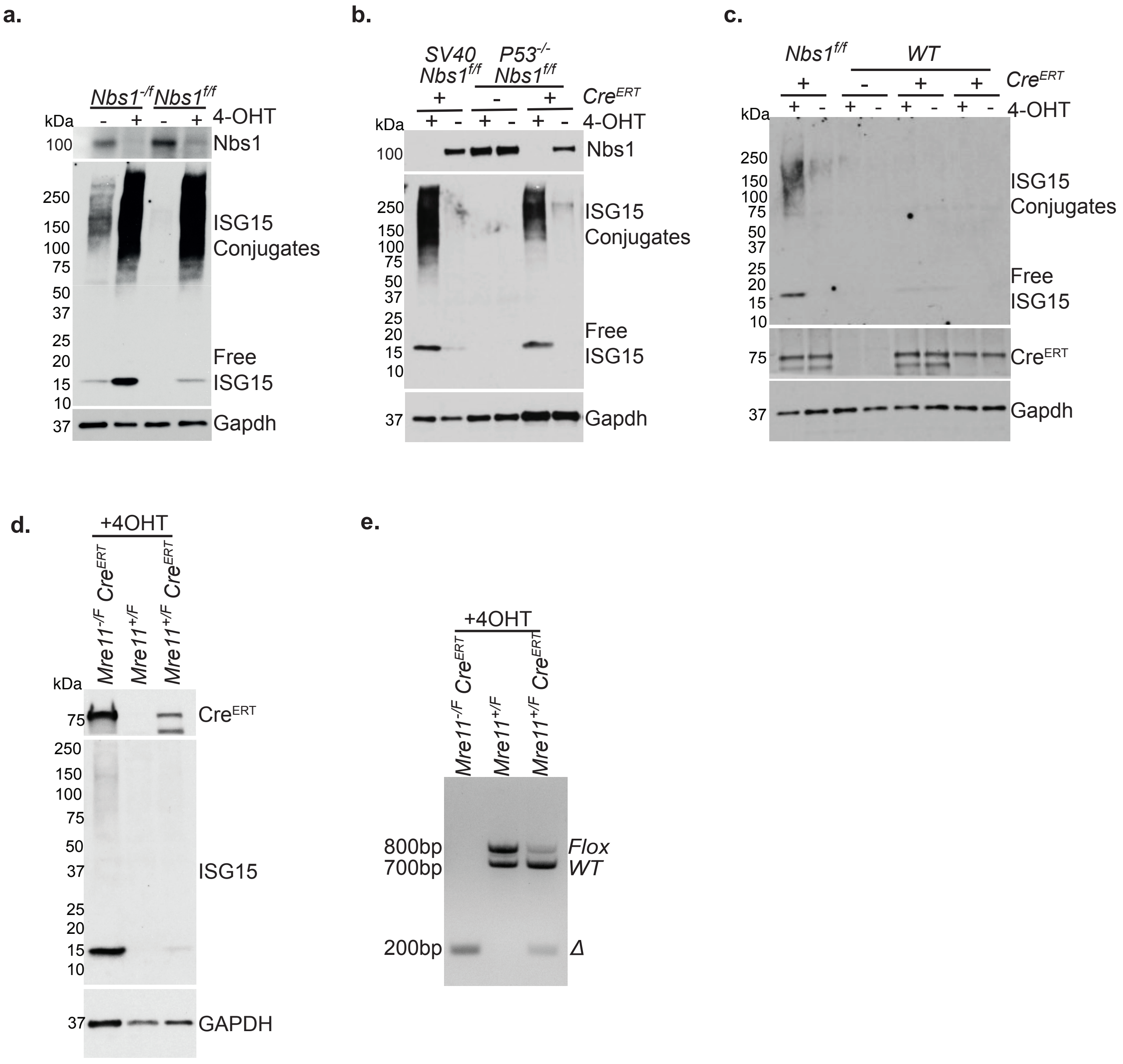

Supplementary Fig. 3. Mre11 complex ISGylation Controls

a. Western blot of whole cell extracts with or without induction of *Nbs1* deletion **b.** Western blot of whole cell extracts taken from MEFs immortalized via SV40 infection or *p53* deletion, with or without stable expression of Cre^{ERT} +/- 4-OHT addition. **c.** Western blot of whole cell extracts from *WT* MEFs with or without stable expression of Cre^{ERT} from two seperate constructs, +/- 4-OHT addition. **d.** Western blot of whole cell extracts from the indicated cell lines after 4-OHT addition. **e.** PCR genotyping of Mre11 exon 5 using primers characterised in Buis et al., 2008 from the indicated genotypes after 4-OHT treatment. DNA genotyping samples were extracted from the same cells as the whole cell extracts in d.

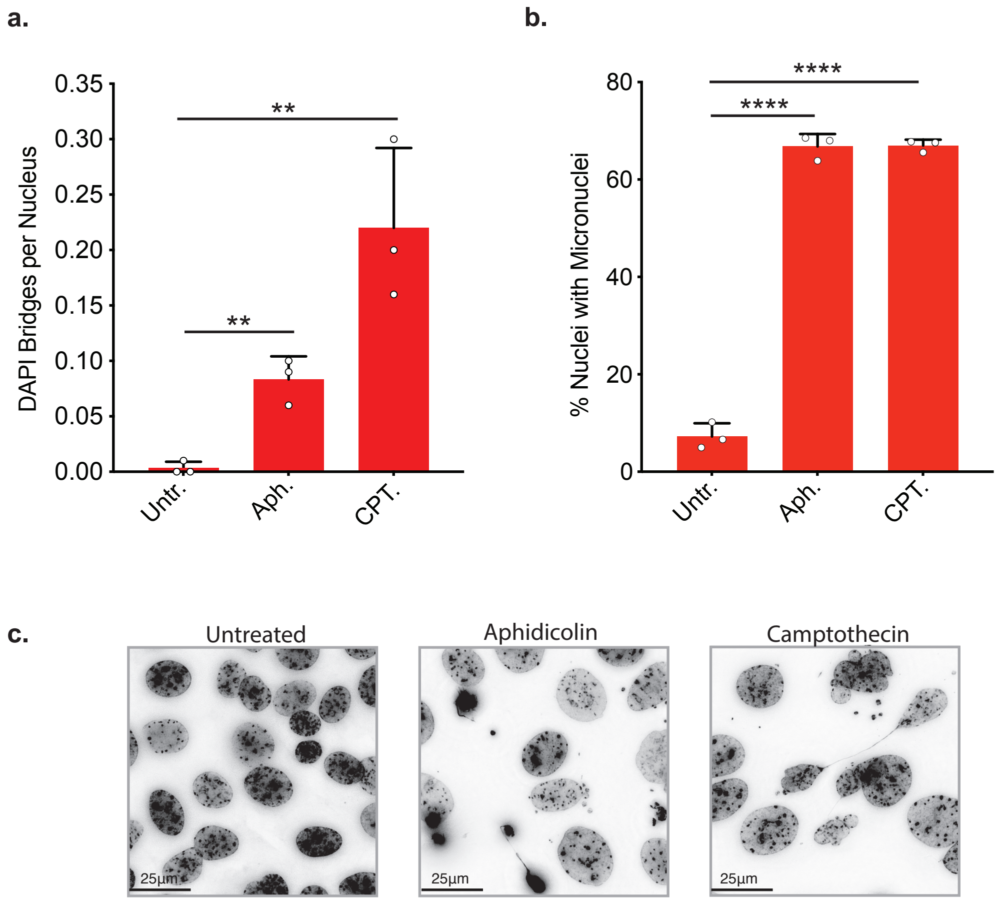

Supplemenary Fig. 4. Replication Stress Induces Micronuclei and DNA Bridges

a. Quantification of cells with micronuclei upon 24 hrs treatment with 1 μM Aphidicolin (Aph) or 0.25 μM Camptothecin (CPT). Mean of three independent experiments with SD shown. Stars reperesent significance via two-tailed unpaired *t*-test, Aph $p= 0.0030$, CPT $p= 0.0066$. **b.** DAPI stained DNA bridges seen upon 24 hr treatment with 1 μM Aph or 0.25 μM CPT. Stars reperesent significance via two-tailed unpaired *t*-test, Aph $p= < 0.0001$, CPT $p= < 0.0001$.**c.** Example images for the data shown in panels a. and b. Between 74 and 181 cells counted per condition per repeat (Untr. n= 449; Aph n= 247; CPT n= 254) .

a.

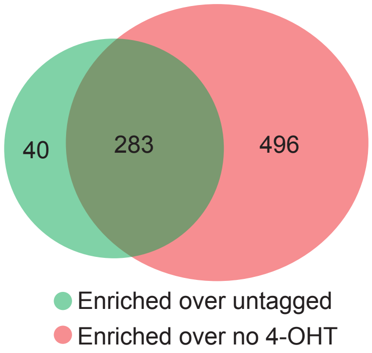

b.

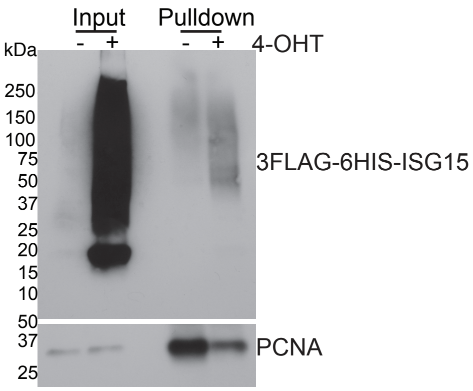

c.

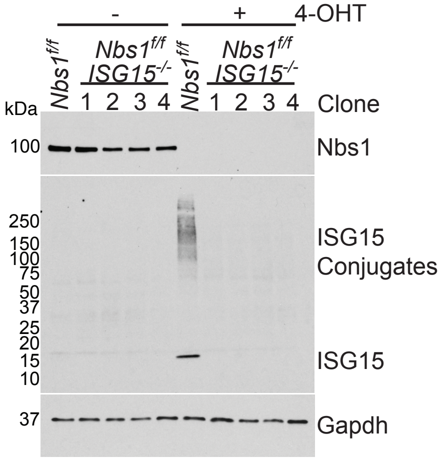

d.

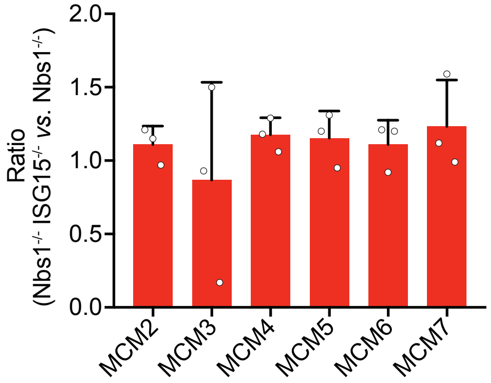

E.

Protein	Log2	Protein	Log2
Nipbl	-0.62	Ercc4	-1.29
Xrcc5	-0.63	Rnaseh2a	-1.30
ATM	-0.65	Atfip	-1.32
Sumo1	-0.66	Rad54L2	-1.41
Polb	-0.67	Zranb3	-1.45
Parp3	-0.71	Mcmbp	-1.46
Kat7	-0.71	Dnmt1	-1.47
Trim28	-0.73	Topbp1	-1.53
Ncapd3	-0.76	Esco2	-1.55
Rif1	-0.76	Hmces	-1.61
Chd1l	-0.76	Polk	-1.73
Sumo2	-0.78	Exo1	-1.74
Plk1	-0.83	Ncaph1	-1.75
Cdkn2aip	-0.85	Fam175a	-1.75
Fancd2	-0.86	Wrnip1	-1.76
Fanci	-0.94	Brcc3	-1.78
Blm	-0.94	Kdm1a	-1.83
Timeless	-0.95	Tipin	-1.98
Obc1	-0.98	Mlh1	-1.99
Wapal	-0.99	Hmgb3	-1.99
Cdyl	-1.00	Smarca1	-2.00
Smarcal1	-1.00	Clspn	-2.00
Ercc3	-1.02	Cdk2	-2.00
Atrx	-1.03	Mdc1	-2.07
Cul4a	-1.03	Setdb1	-2.46
Smarcad1	-1.03	Bard1	-2.46
Tonsl	-1.19	Ercc1	-2.46

Supplementary Fig. 5. ISG15 Acts at Replication Forks

a. Venn diagram relating to Figures 4C (green) and Figure 4D (pink) comparing proteins at least two-fold enriched in 3xFLAG-6HIS-ISG15 pulldowns over the indicated control. Data shown in Supplementary Table 3. **b.** iPOND followed by western blotting in *3FLAG-6HIS-ISG15 Nbs1$^{f/f}$* Mefs with or without 4-OHT induced *Nbs1* deletion. **c.** Western blot of whole cell extracts from *ISG15$^{-/-}$ Nbs1$^{f/f}$* MEFs with or without 4-OHT induced *Nbs1* deletion. Four independent *ISG15$^{-/-}$ Nbs1$^{f/f}$* clones shown. **d.** MCM control for the iPOND experiment shown in Figure 4G. Mean of three independent experiments with SD. **e.** Table showing DNA replication/repair proteins depleted on nascent DNA in *Nbs1$^{-/-}$ ISG15$^{-/-}$* compared to *Nbs1$^{-/-}$*. Data associated with Figure 4G and Supplementary Table 4. Mean Log2 ratios from three independent repeats shown.

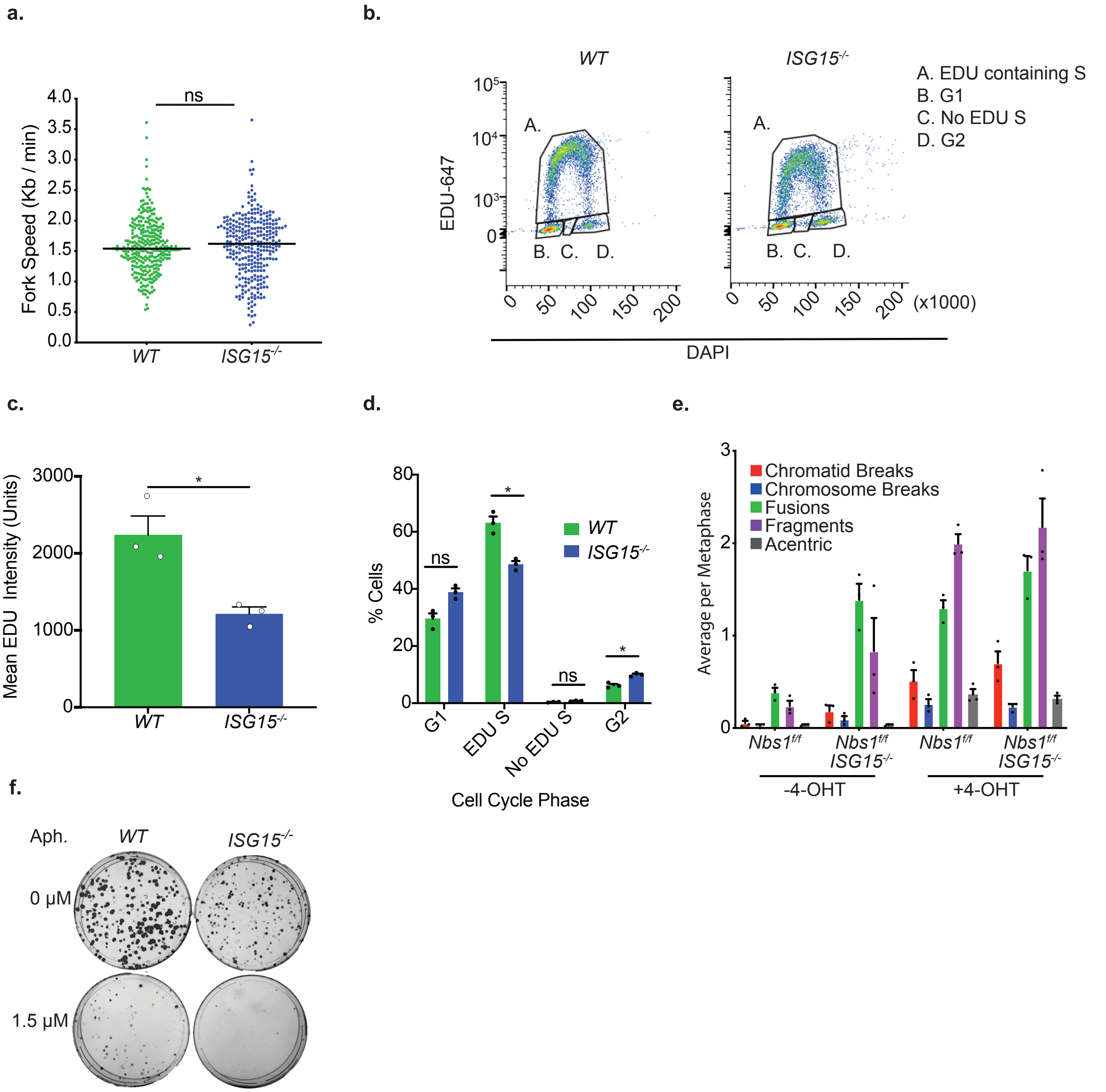

Supplementary Fig. 6. *ISG15*$^{-/-}$ MEFs Exhibit Genomic Instability

a. DNA combing analysis of the indicated cell lines after a 30 min IdU pulse followed by a 30 min CldU pulse. CldU tract length was measured, and fork velocity calculated. Data from three independent experiments combined. Median velocity shown. Two tailed Mann-Whitney test, p= 0.3783 (*WT* n=304, *ISG15*$^{-/-}$ n= 301) . **b.** Example EDU-flow cyctomerty graphs of the indicated *ISG15* genotypes. Boxes represent gating for cell cycle phases. **c.** Quantification of average EDU intensities from 6b. Mean of three independent experiments with SEM. Two-tailed unpaired *t*-test, p= 0.0182. **d.** Quantification of cell cycle phases from 6b. Mean of three independent experiments with SEM. Two-tailed paired *t*-test, G1 p = 0.0903, EDU-S p = 0.0407, No EDU-S p = 0.0747, G2 p = 0.0351. **e.** Genomic aberrations broken down by aberration type for the metaphase spread data shown in Figs. 5F and G. Mean of three independent experiments with SEM. **f.** Example images of the colony forming data shown in Fig. 5H. *WT* or *ISG15*$^{-/-}$ MEFs colonies 10 days after 16 hrs treatment with the indicated dose of aphidicolin (Aph.)
