# Supplementary material for: ISG15 conjugation to proteins on nascent DNA mitigates DNA replication stress
Source: Nat Commun. 2022 Oct 10;13:5971. doi: 10.1038/s41467-022-33535-y (PMC9550767; doi:10.1038/s41467-022-33535-y)
Supplement: Supplementary file 2 — Description of Additional Supplementary Files [file 41467_2022_33535_MOESM2_ESM.pdf]

Description of Additional Supplementary Files

File Name: Supplementary Data 1
Description: **Pulse-chase iPOND-SILAC-MS data. Associated with Fig. 1d, Supplementary Fig. 2 a, b.** Data includes normalized heavy *vs.* light (H/L) ratios for proteins enriched on nascent DNA in each of the three repeats, mean normalized H/L ratios, mean normalized Log2 H/L ratios and -Log *p*-values (unpaired *t*-test). GO terms with *p*-values (hypergeometric distribution) for the identified proteins are also displayed. Data underlying the Venn diagrams in Supplementary Fig. 2b are included as separate tabs.

File Name: Supplementary Data 2
Description: **iPOND-SILAC-MS data from *Nbs1$^{f/f}$* cells with or without 4-OHT treatment. Associated with Fig. 1e.** Data includes normalized heavy *vs.* light (H/L) ratios, mean normalized H/L ratios, normalized Log2 H/L ratios and -Log *p*-values (unpaired *t*-test) for proteins identified in each of the three repeats. Lists of GO terms with *p*-values (hypergeometric distribution) for proteins identified as enriched or depleted on nascent DNA upon Nbs1depletion are included as separate tabs.

File Name: Supplementary Data 3
Description: **TMT-Mass spectrometry data of chromatin associated ISGylated proteins. Data accompanies Fig. 4b-d and Supplementary Fig. 5a.** Data includes mean Log2 ratios and mean Log10 absorbances for *Nbs1$^{-/-}$ 3FLAG-6HIS-ISG15 vs. Nbs1$^{-/-}$ ISG15* (Fig. 4c) and *Nbs1$^{-/-}$ 3FLAG-6HIS-ISG15 vs. Nbs1$^{+/+}$ 3FLAG-6HIS-ISG15* (Fig. 4d). Lists of GO terms with p-values (hypergeometric distribution) for the identified proteins are also included. Data underlying the Venn diagram in Supplementary Figure 5a are displayed in a separate tab.

File Name: Supplementary Data 4
Description: **iPOND-SILAC-MS data from *Nbs1$^{-/-}$ vs. Nbs1$^{-/-}$ ISG15$^{-/-}$*. Data associated with Fig. 4g. and Supplementary Fig. 5d.** Data includes normalized heavy *vs.* light (H/L) ratios, mean normalized H/L ratios, and mean normalized Log2 H/L ratios for proteins identified in each of the three repeats. GO terms with *p*-values (hypergeometric distribution) for the identified proteins are displayed in separate tabs.

File Name: Supplementary Data 5
Description: **Reagent tables.** Lists of antibodies, qPCR primers, CRISPR guides and templates and genotyping primers used in this study.
