# Supplementary material for: ISG15 conjugation to proteins on nascent DNA mitigates DNA replication stress
Source: Nat Commun. 2022 Oct 10;13:5971. doi: 10.1038/s41467-022-33535-y (PMC9550767; doi:10.1038/s41467-022-33535-y)
Supplement: Supplementary file 8 — Reporting Summary [file 41467_2022_33535_MOESM8_ESM.pdf]

Corresponding author(s): John HJ Petrini

Last updated by author(s): Aug 29, 2022

Reporting Summary

Nature Portfolio wishes to improve the reproducibility of the work that we publish. This form provides structure for consistency and transparency in reporting. For further information on Nature Portfolio policies, see our Editorial Policies and the Editorial Policy Checklist.

Statistics

For all statistical analyses, confirm that the following items are present in the figure legend, table legend, main text, or Methods section.

n/a	Confirmed	
☐	☒	The exact sample size (*n*) for each experimental group/condition, given as a discrete number and unit of measurement
☐	☒	A statement on whether measurements were taken from distinct samples or whether the same sample was measured repeatedly
☐	☒	The statistical test(s) used AND whether they are one- or two-sided<br>*Only common tests should be described solely by name; describe more complex techniques in the Methods section.*
☒	☐	A description of all covariates tested
☒	☐	A description of any assumptions or corrections, such as tests of normality and adjustment for multiple comparisons
☐	☒	A full description of the statistical parameters including central tendency (e.g. means) or other basic estimates (e.g. regression coefficient) AND variation (e.g. standard deviation) or associated estimates of uncertainty (e.g. confidence intervals)
☐	☒	For null hypothesis testing, the test statistic (e.g. *F*, *t*, *r*) with confidence intervals, effect sizes, degrees of freedom and *P* value noted<br>*Give P values as exact values whenever suitable.*
☒	☐	For Bayesian analysis, information on the choice of priors and Markov chain Monte Carlo settings
☒	☐	For hierarchical and complex designs, identification of the appropriate level for tests and full reporting of outcomes
☒	☐	Estimates of effect sizes (e.g. Cohen's *d*, Pearson's *r*), indicating how they were calculated

Our web collection on statistics for biologists contains articles on many of the points above.

Software and code

Policy information about availability of computer code

Data collection	No software was used for data collection
Data analysis	For graph creation and statistical analysis: GraphPAD-Prism version 9, Microsoft Excel version 16.54<br>For flow cytometry diagrams and analysis: FlowJO version 10.7,<br>For image analysis: Fiji (ImageJ) version 2.0,<br>For mass spec data analysis: MaxQuant version 1.5.3.30, Proteome DIscoverer (PD) version 2.4.1.15 (Thermo-Sceintific) searched with the SEQUEST HT search engine with a Uniprot protein database downloaded on 2019/12/13 (92,249 entries).<br>For guide RNA design: Benchling (https://www.benchling.com), CHOPCHOP (https://chopchop.cbu.uib.no).<br>For Venn diagram creation: https://bioinformatics.psb.ugent.be/webtools/Venn/<br>For GO terms: http://www.informatics.jax.org

For manuscripts utilizing custom algorithms or software that are central to the research but not yet described in published literature, software must be made available to editors and reviewers. We strongly encourage code deposition in a community repository (e.g. GitHub). See the Nature Portfolio guidelines for submitting code & software for further information.

Data

Policy information about availability of data

All manuscripts must include a data availability statement. This statement should provide the following information, where applicable:

- Accession codes, unique identifiers, or web links for publicly available datasets
- A description of any restrictions on data availability
- For clinical datasets or third party data, please ensure that the statement adheres to our policy

Data plotted in the mass spec graphs, Venn diagrams and tables (Figures 1D-E, 4 C-D, 4G, Supplementary Figures 2 A-B, 2E, 5A, 5C-D) are presented in the supplementary data.
The raw mass spec data have been deposited to the ProteomeXchange Consortium via the PRIDE partner repository with the dataset identifier PXD031770
Original un-cropped scans of western blots are displayed in the Source Data file as is the data plotted in each of the graphs

Field-specific reporting

Please select the one below that is the best fit for your research. If you are not sure, read the appropriate sections before making your selection.

☒ Life sciences ☐ Behavioural & social sciences ☐ Ecological, evolutionary & environmental sciences

For a reference copy of the document with all sections, see nature.com/documents/nr-reporting-summary-flat.pdf

Life sciences study design

All studies must disclose on these points even when the disclosure is negative.

Sample size	Sample sizes were chosen according to standards within the field.<br>For metaphase spreads a total of ~150 spreads were counted per condition across three biological repeats. This is consistent with previously published numbers e.g. Kim et al., 2017; Balestrini et al., 2016,<br>For DNA combing >200 fibers were counted for fork velocity per genotype across 3 biological repeats. For for fork symmetry >80 fibers were counted per genotype across 3 biological repeats. Total ssDNA counter staining was used to increase data accuracy. These numbers were determined in Techer et al., 2013<br>Sample size information for each experiment is described in the associated figure legend and/or methods section
Data exclusions	For iPOND SILAC-Mass Spec experiments, only proteins that were identified in at least 2 out of the 3 experiments were plotted on the graphs and shown in corresponding supplementary data tables. This is to ensure only reproducible hits are displayed and to reduce the risk of false positive hits. This strategy was pre-established before analyzing the data to avoid bias. Full data sets have been deposited on the ProteomeXchange Consortium
Replication	Experiments were performed at least in triplicate, except for experiments stated to be repeated in duplicate. Individual points for each repeat are shown on all bar charts. All repeats gave similar results.
Randomization	Samples were randomly assigned as control or treatment groups
Blinding	For all microscopy experiments slides were blinded for acquisition counting and analysis.<br>For all other experiments blinding was not applicable as the data was obtained and presented in an unbiased manner such as via western blotting or SILAC-Mass Spec

Reporting for specific materials, systems and methods

We require information from authors about some types of materials, experimental systems and methods used in many studies. Here, indicate whether each material, system or method listed is relevant to your study. If you are not sure if a list item applies to your research, read the appropriate section before selecting a response.

Materials & experimental systems

n/a	Involved in the study	
☐	☒	Antibodies
☐	☒	Eukaryotic cell lines
☒	☐	Palaeontology and archaeology
☒	☐	Animals and other organisms
☒	☐	Human research participants
☒	☐	Clinical data
☒	☐	Dual use research of concern

Methods

n/a	Involved in the study	
☒	☐	ChIP-seq
☐	☒	Flow cytometry
☒	☐	MRI-based neuroimaging

Antibodies

Antibodies used	Antibody's used are also shown in Supplementary Table 5<br>Nbs1 CST 3002 1:1000<br>Chk1 CST 2360 1:1000<br>pChk1 pS345 CST 2348 1:1000<br>H2aX Bethyl A300-083A 1:20000<br>yH2ax pS129 Abcam ab-2983 1:2000<br>Kap1 (23) Santa Cruz SC-136102 1:5000<br>pKap1 pS824 Abcam ab-70369 1:5000<br>Rpa32 (4E4) CST 2208 1:10000<br>pRpa32 pS4/8 Bethyl A300-245A 1:1000<br>Gapdh (6C5) Santa Cruz sc-32233 1:1000<br>Actin-Hrp Abcam ab-49900 1:30000<br>H3 Abcam ab-1791 1:400000<br>Isg15 (E-9) Santa Cruz sc-166794 1:100<br>Pcna (PC10) CST 2586 1:1000<br>Tbk1 (D1B4) CST 3504 1:1000<br>pTbk1 pS17) CST 5483 1:500<br>pSTING pS365 CST 72971 1:1000<br>cGAS (D3080) CST 31659 1:1000<br>FLAG M2 Sigma F1804 1:1000<br>Top2A Santa Cruz sc-365916 1:100<br>Smc3 CST 5696 1:1000<br>VCP CST 2684 1:200<br>Fen1 Bethyl A300-255A 1:1000<br>CRE Millipore 69050 1:10000<br>Mre11 In house N/A 1:10000<br>FLAG-HRP Sigma A8592 1:750<br>Rabbit anti-Mouse Hrp Sigma-Aldrich AP160P 1:10000<br>Goat anti-Rabbit Hrp Sigma-Aldrich AP156P 1:10000<br>BrdU for CldU Abcam ab6326 1:5<br>BrdU (B44) for IdU BD 347580 1:10<br>ssDNA Millipore (16-19) MAB3034 1:20<br>Goat anti-mouse Alexafluor 488 plus Life Technologues A32723 1:100<br>Goat anti-Rat Alexafluor 568 Life Technologues A11077 1:100<br>Goat anti-mouse Alexafluor 647 Life Technologues A21236 1:100
Validation	Nbs1 CST 3002 by manufacturer https://www.cellsignal.com/products/primary-antibodies/p95-nbs1-antibody/3002 (40 citations) and by us via use of an inducible Nbs1 knockout cell line and western blotting in Figures 1A, 2A, 3F, 5A, SD2, S3 A-B, S5B<br>Chk1 CST 2360 by manufacturer https://www.cellsignal.com/products/primary-antibodies/chk1-2g1d5-mouse-mab/2360 (389 citations)<br>pChk1 pS345 CST 2348 https://www.cellsignal.com/products/primary-antibodies/phospho-chk1-ser345-133d3-rabbit-mab/2348 (708 citations) and by us via use of CPT and APH treated positive controls in Figure 1A.<br>H2aX Bethyl A300-083A by manufacturer https://www.fortislife.com/products/primary-antibodies/rabbit-anti-h2ax-antibody/BETHYL-A300-083 (8 citations)<br>yH2ax (pS129) Abcam ab-2983 by manufacturer https://www.abcam.com/gamma-h2ax-phospho-s139-antibody-ab2893.html (321 citations) and by us via use of CPT and APH treated positive controls in Figure 1A<br>Kap1 (23) Santa Cruz SC-136102 by manufacturer https://www.scbt.com/p/tif1beta-antibody-23<br>pKap1 pS824 Abcam ab-70369 by manufacturer https://www.abcam.com/kap1-phospho-s824-antibody-ab70369.html (46 citations) and by us via use of CPT and APH treated positive controls in Figure 1A<br>Rpa32 (4E4) CST 2208 by manufacturer https://www.cellsignal.com/products/primary-antibodies/rpa32-rpa2-4e4-rat-mab/2208?site-search-type=Products&N=4294956287&Ntt=2208+&fromPage=plp&_requestid=1138277 (123 citations)<br>pRpa32 pS4/8 Bethyl A300-245A https://www.fortislife.com/products/primary-antibodies/rabbit-anti-phospho-rpa32-s4-s8-antibody/BETHYL-A300-245 (106 citations) and by us via use of CPT and APH treated positive controls in Figure 1A<br>Gapdh (6C5) Santa Cruz sc-32233 by manufacturer https://www.scbt.com/p/gapdh-antibody-6c5?requestFrom=search (3,956 citations)<br>Actin-Hrp Abcam ab-49900 by manufacturer https://www.abcam.com/hrp-beta-actin-antibody-ac-15-ab49900.html (322 citations)<br>H3 Abcam ab-1791 by manufacturer https://www.abcam.com/histone-h3-antibody-nuclear-marker-and-chip-grade-ab1791.html (3819 citations)<br>Isg15 (E-9) Santa Cruz sc-166794 by manufacturer https://www.scbt.com/p/isg15-antibody-e-9?requestFrom=search and by us via CRISPR knockout and western blotting in Figure S5B<br>Pcna (PC10) CST 2586 by manufacturer https://www.cellsignal.com/products/primary-antibodies/pcna-pc10-mouse-mab/2586 (495 citations)<br>Tbk1 (D1B4) CST 3504 by manufacturer https://www.cellsignal.com/products/primary-antibodies/tbk1-nak-d1b4-rabbit-mab/3504 (322 citations)<br>pTbk1 pS17) CST 5483 by manufacturer https://www.cellsignal.com/products/primary-antibodies/phospho-tbk1-nak-ser172-d52c2-xp-rabbit-mab/5483?site-search-type=Products&N=4294956287&Ntt=5483+&fromPage=plp&_requestid=1139904 (509 citations)<br>pSTING pS365 CST 72971 by manufacturer https://www.cellsignal.com/products/primary-antibodies/phospho-sting-ser365-d8f4w-rabbit-mab/72971 (37 citations)<br>cGAS (D3080) CST 31659 by manufacturer https://www.cellsignal.com/products/primary-antibodies/cgas-d3o8o-rabbit-mab-mouse-specific/31659 (76 citations)<br>FLAG M2 Sigma F1804 by manufacturer https://www.sigmaaldrich.com/US/en/product/sigma/f1804 and by us via western blotting of untagged controls in Figures 4a

Top2A Santa Cruz sc-365916 by manufacturer https://www.scbt.com/p/topo-iialpha-antibody-f-12?requestFrom=search (16 citations)
Smc3 CST 5696 by manufacturer https://www.cellsignal.com/products/primary-antibodies/smc3-d47b5-rabbit-mab/5696?site-search-type=Products&N=4294956287&Ntt=5696+&fromPage=plp&_requestid=1127428 (11 citations)
VCP CST 2684 by manufacturer https://www.cellsignal.com/products/primary-antibodies/ikkb-antibody/2684 (130 citations)
Fen1 Bethyl A300-255A by manufacturer https://www.fortislife.com/products/primary-antibodies/rabbit-anti-fen1-antibody/BETHYL-A300-255
CRE Millipore 69050 by manufacturer https://www.emdmillipore.com/US/en/product/Anti-Cre-Antibody,EMD_BIO-69050 an by us via western blotting from cells expressing a CRE cassette in Figure S3C.
Mre11 In house previously validated in Kim et al., 2017; Kim etal., 2019; Sracker et al., 2007; Theunissen et al., 2003 etc. It was further validated using Mre11 inducible knockout MEFs and western blotting in this study Figure 2C.
FLAG-HRP Sigma A8592 by manufacturer https://www.sigmaaldrich.com/US/en/product/sigma/a8592?gclid=EAIaIQobChMIqZXR6a3R-QIViv3jBx1-Tg5wEAAYASAAEgKmWvD_BwE and by us via western blotting an untagged control in Figures 4E-F
Rabbit anti-Mouse Hrp Sigma-Aldrich AP160P by manufacturer https://www.sigmaaldrich.com/US/en/product/mm/ap160p
Goat anti-Rabbit Hrp Sigma-Aldrich AP156P by manufacturer https://www.sigmaaldrich.com/US/en/product/mm/ap156p
BrdU for CldU Abcam ab6326 https://www.abcam.com/brdu-antibody-bu175-icr1-proliferation-marker-ab6326.html and via moleclualr combing manufacturers prtocol.
BrdU (B44) for IdU BD 347580 by manufacturer https://www.fishersci.com/shop/products/anti-brdu-bromodeoxyuridine-clone-bd/BDB347580 and in the Molecular Combing manufacturers protocol
ssDNA Millipore (16-19) MAB3034 by manufacturer https://www.emdmillipore.com/US/en/product/Anti-DNA-Antibody-single-stranded-clone-16-19,MM_NF-MAB3034 and in the Molecular Combing manufacturers protocol
Goat anti-mouse Alexafluor 488 plus Life Technologues A32723 by manufacturer https://www.thermofisher.com/antibody/product/Goat-anti-Mouse-IgG-H-L-Highly-Cross-Adsorbed-Secondary-Antibody-Polyclonal/A32723 (616 citations)
Goat anti-Rat Alexafluor 568 Life Technologues A11077 by mamnufacturer https://www.thermofisher.com/antibody/product/Goat-anti-Mouse-IgG-H-L-Highly-Cross-Adsorbed-Secondary-Antibody-Polyclonal/A32723 (562 citations)
Goat anti-mouse Alexafluor 647 Life Technologues A21236 by manufacturer https://www.thermofisher.com/antibody/product/Goat-anti-Mouse-IgG-H-L-Highly-Cross-Adsorbed-Secondary-Antibody-Polyclonal/A-21236 (820 citations)

Further citations and validation for the commercial antibodies used can be found at citeab https://www.citeab.com

Eukaryotic cell lines

Policy information about cell lines

Cell line source(s)

Nbs1F/F MEFs were previously published (Kim et al., 2017; Kim et al., 2019) and were originally derived from mice kindly gifted by the Zhao-QI Wang (Leibniz Institute for Age Research) (Demuth et al., 2004). Nbs1-/f MEFs were a kind gift from Titia De Lange (Rockefeller University) (Dimitrova and De Lange 2009). Mre11-/F MEFs originated from mice derived in David Ferguson's lab (University of Michigan) (Buis et al., 2008). ATM-/- MEFs originated from mice derived in the Anthony Winshaw-Boris Laboratory (Case Western Reserve Univsity) (Barlow et al., 1996). Nbs1F/F ISG15-/-, Nbs1F/F cGAS-/-, Nbs1f/f Usp18-/- , Nbs1f/f 3FLAG-6HIS-ISG15 MEFs were created in this study via CRISPR-CAS9 editing of NBS1F/F MEFs.

Authentication

Nbs1F/F, Nbs1-/F, Mre11F/F and ATM-/- MEFs were all previously published and authenicated.
For example: Nbs1F/F (Kim et al., 2017; Kim et al., 2019). Nbs1-/F (Dimitrova and De Lange 2009; Kim et al., 2017). Mre11-/F (Buis et al., 2008). ATM-/- MEFs (Barlow et al., 1996; Morales et al., 2005). They were further authenticated in this manuscript via western blotting.
Cell lines derived for this manuscript via CRISPR-CAS9 editing were genotyped via PCR followed by restriction digest and agarose gel electrophoresis, via sequencing, via ICE analysis (Synthego) and via western blotting

Mycoplasma contamination

Cell lines were tested monthly via PCR for mycoplasma and tested negative

Commonly misidentified lines
(See ICLAC register)

No commonly misidentified cell lines were used in this study

Flow Cytometry

Plots

Confirm that:

☒ The axis labels state the marker and fluorochrome used (e.g. CD4-FITC).

☒ The axis scales are clearly visible. Include numbers along axes only for bottom left plot of group (a 'group' is an analysis of identical markers).

☒ All plots are contour plots with outliers or pseudocolor plots.

☒ A numerical value for number of cells or percentage (with statistics) is provided.

Methodology

Sample preparation

Cells were pulsed for 30 mins with 15 µM EDU and samples prepared using the Click-iT Plus EdU Alexa Fluor 647 Flow Cytometry Assay Kit (Thermo Fisher #C10634) according to manufactures instructions. Total DNA was stained with FxCycle Violet Stain (Thermo Fisher Scientific, F10347).

Instrument	Fortessa 3
Software	FlowJo
Cell population abundance	10,000 cells were analyzed per sample
Gating strategy	Live single cells were gated and cell cycle populations analyzed according to gating in Supplementary Figures 1B and 6B. Doublet removal gating strategy is provided in the Source Data file.

☐ Tick this box to confirm that a figure exemplifying the gating strategy is provided in the Supplementary Information.
